# The effect of the APOE4 genotype on physiological and cognitive health in randomised controlled trials with an exercise intervention: a systematic review and meta-analysis

**DOI:** 10.1186/s13063-024-08696-4

**Published:** 2025-01-20

**Authors:** Felicity S. E. Spencer, Richard J. Elsworthy, Leigh Breen, Jon R. B. Bishop, Connor Dunleavy, Sarah Aldred

**Affiliations:** 1 School of Sport, Exercise and Rehabilitation Sciences, University of Birmingham, Birmingham, B15 2TT UK; 2 Birmingham Clinical Trials Unit, Institute of Applied Health Research, Public Health Building, College of Medical and Dental Sciences, University of Birmingham, Birmingham, UK

**Keywords:** Exercise, Alzheimer’s disease, Randomised controlled trials

## Abstract

**Background:**

Alzheimer’s disease is caused by modifiable and non-modifiable risk factors. Randomised controlled trials have investigated whether the strongest genetic risk factor for Alzheimer’s disease, APOE4, impacts the effectiveness of exercise on health. Systematic reviews are yet to evaluate the effect of exercise on physical and cognitive outcomes in APOE genotyped participants. A quality assessment of these randomised controlled trials is needed to understand the impact genotype has on the potential success of intervention. This systematic review aimed to determine if the APOE4 genotype influences the effectiveness of exercise-based randomised controlled trials.

**Method:**

Searches on MEDLINE, EMBASE, and PsycINFO identified eligible exercise based randomised controlled trials incorporating participants with varied cognitive abilities. Quality assessments were conducted.

**Results:**

Nineteen studies met the inclusion criteria for systematic review, and 3 for the meta-analysis. Very low to moderate quality evidence showed that APOE4 carriers benefitted more than APOE4 non-carriers on cognitive (e.g. executive function, learning) and physical (e.g. relative telomere length) outcomes after exercise; and that APOE4 non-carriers benefited over carriers for physical (serum BDNF, gait speed) and cognitive (global cognition, verbal memory) markers. Very low quality evidence indicated that there was no evidence of difference between APOE4 carriers and non-carriers on physical function outcomes in meta-analysis. Several areas of study design and reporting, including maintenance of relative exercise intensity and complete statistical reporting, were identified as needing improvement.

**Discussion:**

This systematic review found very limited evidence to suggest that exercise interventions can benefit APOE4 carriers and non-carriers equally, though conclusions were limited by evidence quality. Further randomised controlled trials, stratifying participants by APOE status are required to better understand the relationship between APOE genotype and the effect of exercise on health-related outcomes.

**Trial registration:**

This review was registered with PROSPERO (CRD42023436842). Registered on June 16, 2023.

**Supplementary Information:**

The online version contains supplementary material available at 10.1186/s13063-024-08696-4.

## Introduction

Alzheimer’s disease (AD) is the most common cause of dementia, resulting in up to 7 million new diagnoses of dementia every year [1]. Characterised by the accumulation of amyloid plaques and neurofibrillary tau tangles in the brain, AD is a debilitating neurodegenerative condition. Genetic and lifestyle factors contribute to the development of AD; with up to 40% of the risk of dementia caused by modifiable lifestyle factors [2]. Therefore, developing optimal lifestyle interventions for those at risk of AD is important to reduce the significant financial and societal burden of AD [3].

A lack of physical activity is a key modifiable risk factor for AD [4], as over a quarter of the world’s population aged 18 and over do not achieve sufficient activity levels every week [5]. It is well-established that regular physical activity reduces the risk of AD [6]. This is likely to be the result of physical activity directly affecting changes in the brain and body associated with the development of AD. This includes a direct effect on amyloid processing [7]; in addition to oxidative stress [8, 9] and neuroinflammation [9, 10]. Physical activity further reduces the risk of AD through beneficial downstream effects on other risk factors for dementia, such as obesity and hypertension [11, 12].

The strongest genetic risk factor for late-onset AD is the presence of the ɛ4 variant of the Apolipoprotein E (APOE) protein. APOE is primarily involved in the metabolism and transport of lipids and cholesterol; but also plays an important role in neuronal maintenance and repair [13]. There are three main alleles of the gene (ɛ2, ɛ3, ɛ4); each affecting the risk of chronic illness [14, 15]. In particular, the presence of APOE4 is associated with an increased risk of dementia [16, 17], and more specifically AD. This risk increases dependent on the exact APOE status. For instance, carrying one APOE4 allele doubles or triples the risk of being diagnosed with AD; and homozygous carriers have an eight times higher likelihood of being diagnosed with AD than non-carriers [18].

Carriers of APOE4 are more likely to experience changes in the brain that may be precursor to AD, even if they never develop AD itself. Carrying APOE4 is associated with an increased burden of amyloid-β, even in cognitively normal adults [19–21]. The effect of APOE4 on neurofibrillary tau tangles is less established, as contradictory studies using positron emission topography (PET) scans show that APOE4 is both related [22] and unrelated [23] to tau presence [21]. Furthermore, brain structure is also affected by APOE4. Carrying APOE4 is associated with increased white matter hyperintensities; alongside smaller hippocampal volume [20, 24]. The smaller hippocampal volume in APOE4 homozygotes was associated with worse scores on tests of episodic memory, suggesting that changes in the brain associated with APOE4 occur alongside reduced cognitive function [20]. Taken together, this demonstrates that APOE4 contributes to abnormal changes in the brain, providing a mechanism through which APOE may increase risk of AD.

Physical activity may directly counteract the effect of APOE4 on the brain. Experimental animal research has demonstrated that exercise can decrease levels of amyloid-β, as well as increase levels of amyloid degrading enzymes that are responsible for amyloid clearance [25]. Tau pathology can also be reduced following long-term exercise [26]. In addition to this, the structure of the brain is directly altered by regular physical activity. Large bodies of evidence suggest that hippocampal volume increases following exercise in healthy adults and individuals with MCI [27, 28]. There is also some suggestion that white matter hyperintensities are reduced following aerobic exercise, though the effect may be more pronounced in males than females [29].

The interaction between physical activity, APOE4, and the brain has previously been reviewed. De Frutos-Lucas et al. (2020) [30] reviewed the effect of physical activity on brain health of APOE4 carriers in observational studies. Though the heterogeneity between studies made it difficult to draw strong conclusions; physical activity was more protective for APOE4 carriers than non-carriers in studies with larger sample sizes of APOE4 carriers [30]. There was also an inverse relationship between amyloid burden and exercise in carriers of APOE4, and both carriers and non-carriers showed positive responses to exercise in neuroimaging studies. Cancela-Carral et al. (2021) [31] investigated the impact of APOE4 presence on cognitive outcomes in exercise based randomised controlled trials (RCTs). In contrast to [30], non-carriers of APOE4 benefitted more than APOE4 carriers from exercise interventions carried out at a higher intensity, though both groups could perform this level of exercise. Furthermore, non-carriers of APOE4 showed more cognitive benefit than APOE4 carriers for exercise interventions lasting more than 50 sessions.

RCTs are considered the ‘gold-standard’ of evidence to determine whether an intervention is beneficial [32]. Significant findings from RCTs are considered more conclusive than findings from observational research [33] and can influence public health policy and practice. However, many RCTs with exercise interventions have been conducted with inconclusive results, and it remains unclear whether carriers and non-carriers of APOE4 respond equally to exercise. The diverse results from RCTs may arise from varying levels of quality of the trial and/or intervention methodology across these studies. Previous research attempting to synthesise the results from exercise based RCTs in this area is yet to examine this in sufficient detail.

Current guidance for conducting RCTs with an exercise-based intervention is limited, and successful RCTs are required to maximise their value for money. Furthermore, variation in study design makes it difficult to understand the full benefits of exercise, and the range of outcomes chosen by RCTs has not been systematically examined. Therefore, the main objective of this systematic review and meta-analysis was to assess the level of the evidence as to whether carriers of APOE4 (ɛ3/ɛ4 and ɛ4/ɛ4) and non-carriers of APOE4 (ɛ2/ɛ2, ɛ2/ɛ3, and ɛ3/ɛ3) respond equally to RCTs with an exercise intervention. This systematic review also aimed to determine the quality of these studies and provide recommendation for future exercise based RCTs in this area. To address these objectives, RCTs with an exercise-based intervention conducted in APOE-genotyped adults measuring physical and/or cognitive outcomes were considered for inclusion in this systematic review and meta-analysis.

## Method

### Search strategy

This systematic review was conducted following Preferred Reporting Items for Systematic Reviews and Meta-Analyses (PRISMA) guidelines [34] (see checklist in additional file 1). One author (FS) searched MEDLINE, EMBASE, and PsycINFO from database inception to 19th June 2023 to identify relevant articles. Manual citation searching of articles during full text screening was also conducted.

The search was performed using the following key words: (“APOE” OR “APOE4” OR “Apolipoprotein” or “Apolipoprotein E4” OR “genetic risk” OR “at risk” or “at-risk”) AND (“physical activity” OR “exercise” OR “physical exercise” OR “aerobic exercise” OR “resistance training” OR “strength training” OR “cardiovascular exercise” OR “exercise intervention” OR “running” OR “cycling”) AND (“dementia” OR “Alzheimer’s disease” OR “brain volume” OR “cognition” OR “MRI” OR “EEG” OR “biomarker” OR “BDNF” OR “blood” OR “amyloid” OR “tau” OR “amyloid plaque” OR “tau tangle” OR “brain health” OR “health” OR “quality of life” OR “wellbeing” OR “carer burden”).

Where applicable, search terms were entered into Ovid as key words, as well as subject headings. If there was the opportunity to enter a search term as a heading, then the term was additionally entered as a subject heading to avoid accidentally excluding relevant articles (as in [35]). Once search terms had been entered, retrieved articles were limited by the following OVID categories: (1) English language; (2) “adaptive clinical trial” OR “clinical trial, all” OR “clinical trial, phase i” OR “clinical trial, phase ii” OR “clinical trial, phase iii” OR “clinical trial, phase iv” OR “clinical trial, veterinary” (veterinary clinical trials were included in case of incorrect classification by Ovid) OR “clinical trial protocol” OR “clinical trial” OR “controlled clinical trial” OR “pragmatic clinical trial” OR “randomized controlled trial” OR “randomized controlled trial, veterinary” OR “multicenter study” OR “phase 1 clinical trial” OR “phase 2 clinical trial” OR “phase 3 clinical trial” OR “phase 4 clinical trial”, (3) exclude conference abstracts, meta-analyses and reviews, and (4) journal articles only.

All retrieved studies were imported into Endnote 20.

### Eligibility criteria

Relevant articles were extracted based on the following inclusion and exclusion criteria (Table 1).

**Table 1:** Inclusion and exclusion criteria

Inclusion	Exclusion
RCT, randomised clinical trial, sub study of RCT, pilot RCT	Animal studies
Has a multi-session exercise intervention group	Full text not available
Analyses data by carriers of APOE4 and non-carriers of APOE4 OR between homozygous and heterozygous carriers of APOE4	Conference abstracts, meta-analyses, and reviews
Has a control group that receives nothing that affects the intended outcome measures, e.g. stretch and tone/social activities/treatment as usual	
Conducted in humans	
Conducted in participants over 18 years old	
Written in English	
Quantitative physical or cognitive outcome measure	

Screening was conducted by two authors (FS, RE), and any dispute on eligibility of a given study was resolved by discussion with SA.

Of the articles included in the systematic review; further inclusion criteria were applied to determine inclusion in the meta-analysis. To be included in the meta-analysis, articles must have measured the same outcome, and summary data must have been available post intervention disaggregated by APOE genotype (at least APOE4 carrier vs. APOE4 non-carrier).

### Screening and data extraction

Duplicate articles were identified and excluded. Then article titles and abstracts were screened for eligibility, before full texts were read. At the level of full text reading, the following data was extracted to determine eligibility, and for inclusion in the systematic review: population demographics (mean age, % female, cognitive status); type of study (RCT, sub study of RCT, pilot RCT, or clinical trial); nature of exercise intervention (duration, exercise mode, intensity, frequency, level of supervision, adherence); nature of control intervention; and outcomes (primary and secondary outcomes, measurement tools, and findings). Cognitive (including but not limited to global cognition, executive function, memory etc.) and physical health (including but not limited to gait speed, cardiorespiratory fitness etc.) findings were collected from the latest timepoint of data collection. Following extraction, data was tabulated. Data extraction was conducted by two authors (FS, RE) and attempts were made to contact authors when insufficient data was available (e.g. published data was not disaggregated by APOE4 carrier status).

### Assessment of quality and bias

Quality of the included studies was determined by the CASP RCT Checklist [36], which is a qualitative assessment of study methodology quality with four sections: is the study design valid?; was the study methodologically sound?; what are the results?; do the results help locally?. The Tool for the Assessment of Study qualiTy and reporting in Exercise (TESTEX; [37]) was also used to quantitatively analyse study quality and reporting: 15 points are available in total; 5 for study quality and 10 for study reporting. Briefly, the study quality section assesses the quality of study design, accounting for recruitment and randomisation procedures as well as blinding. The study reporting section focuses on the details reported in the ‘Method’ and ‘Results’ sections, including methods of analysis. FS and RE conducted CASP and TESTEX assessments independently; and where disputes arose, they were resolved by discussion.

The Cochrane Risk of Bias 2 (ROB2) [38] was used to assess bias in five domains: randomisation; deviations from the intended intervention; missing outcome data; measurement of the outcome; and selection of reported results, on the primary outcome of each study. This was independently conducted by FS and RE, and where disputes arose, they were resolved by discussion.

### Data analysis

Outcome scores were synthesised by pre-planned meta-analysis conducted with Review Manager 5.4. Outcome scores (mean/ standard deviation (SD)) at the final time of follow-up were included. Despite their heterogeneity, scores from this timepoint were selected because outcome scores for mean difference between baseline and follow-up could not be calculated for all included articles. As a result of introducing this heterogeneity, and heterogeneity in study design and participant characteristics, random effects models were used [39].

Attempts were made to convert composite *z* scores and standardised scores of outcome measures to the same scale as scores from other trials; though many were not eligible to be included in the meta-analysis due to a lack of available reported statistical information. All outcomes in the meta-analysis were calculated with standardised mean difference [40] unless identical outcome assessments were used between studies.

Where both physical activity and physical activity plus cognitive training conditions were presented, scores from the physical activity condition alone were used. Standard errors (SE) were converted to SD using the following formula [38]:

Standard errors (SE):

$$\text{SD}=\text{SE x }\surd \text{N}$$

Publication bias was examined by the ‘Risk of Bias due to Missing Evidence in a synthesis’ tool (ROB-ME) [39] as this tool is appropriate for assessing bias due to missing evidence regardless of the number of studies selected for inclusion in the meta-analysis. The ROB-ME was completed individually by FS and RE, and any disputes resolved by discussion with SA. Sensitivity analyses were also conducted to determine model uncertainty as a result of bias, by removing studies with some concerns of, and high, risk of bias from the analyses*.* Chi-square and *I*^2^ tests of heterogeneity were conducted to examine the variability between studies. Analysis was planned to examine differences between APOE carriers and non-carriers; however, a lack of data availability meant that this was not feasible for all outcome measures. Therefore, only outcomes relating to physical function (V̇O_2_ max; gait speed; walking endurance and mobility) had sufficient data for synthesis in the meta-analysis.

Two authors (FS and RE) assessed certainty in the body of evidence for each outcome in the systematic review and meta-analysis by GRADE [41]. The authors assessed the outcomes individually and resolved any disputes by discussion. As all included articles were RCTs, randomised clinical trials, sub studies of RCTs, or pilot RCTs, all outcomes began with a starting rating of ‘high quality’, and then were subsequently downgraded by one or two levels for serious or very serious concerns on risk of bias (inadequate methods of sequence generation; lack of allocation concealment; lack of blinding; loss to follow up; failure to follow intention to treat principles; selective outcome reporting; other sources of bias), inconsistency (wide variation in the effect estimates; little or no overlap of confidence intervals associated with effect estimates, statistical tests suggest heterogeneity, whether heterogeneity is explained), indirectness (conclusions did not directly answer research question in terms of population, intervention, comparator, or outcome, or indirect comparison between interventions), imprecision (sample size under 400 for continuous outcomes; precision of effect estimate including upper and lower limits and confidence intervals), or publication bias (number of events in included studies; size of included studies) respectively [41].

## Results

### Search results

**Fig. 1:**
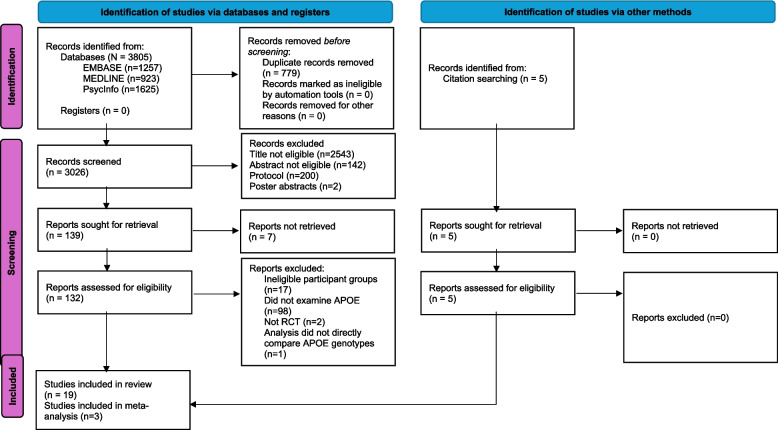
PRISMA flow diagram for the selection and inclusion in the systematic review and meta- analysis

The search identified 3805 potentially relevant papers, which reduced to 3206 once duplicates were excluded. Following the exclusion of irrelevant articles based on titles and abstracts, 139 full texts were assessed for eligibility. After full texts were examined, it was determined that 19 studies were suitable for inclusion in the systematic review (Fig. 1). In these 19 studies, descriptive statistics at baseline and follow-up were rarely disaggregated by APOE genotype, and requests for data were rarely responded to. Due to this lack of data availability, only 3 articles were eligible for inclusion in the meta-analysis. Reasons for exclusion were a lack of studies measuring the same outcomes (many outcomes included in the systematic review were only measured by one study) and a lack of reporting of descriptive data disaggregated by APOE genotype (15/19 provided no data disaggregated by APOE genotype; one study provided this information on request; see additional file 2 for the complete reported descriptive statistics).

### Study characteristics

#### Participants

The included articles in the systematic review (Table 2) have heterogeneous study characteristics.

**Table 2:** Results of the search

Author	Participant characteristics (*N*, mean age, % female, cognitive status)	Exercise schedule	Exercise mode and intensity	Control parameters	Outcome measures	Adherence	Findings	APOE findings
Allard et al. (2017) [42]^c,d^	22 participants Age = 72.0 68.2% female MCI (as defined by [61], Clinical Dementia Rating (CDR) < = 0.5, adjusted Mini Mental State Examination (MMSE) 24–30))	● 20–60 min (progressive) ● 3 days per week ● 24 weeks After first 4–6 weeks, participants include an additional 45–60-min walk, leading to 4 sessions per week	● Treadmill walking/jogging ● Stair stepping ● Elliptical training Initially, sessions lasted 20 min at 50% Volume of Oxygen (V̇O_2_) Max; then increased by 5 min each week until 40 min was reached Then increased by 5% V̇O_2_ Max weekly until achieving 70% V̇O_2_ max Supervised by trained personnel	● 40 min ● 3 days per week ● 24 weeks Static joint stretching targeted at hamstrings, hip flexors, calves, and chests for 15–30 s. Increased number of muscle groups for the first 4 weeks	Baseline 6 months V̇O_2_ Max ● Modified Bruce protocol Serum blood derived neurotrophic factor (BDNF) ● ELISA	Not reported	No significant differences in V̇O_2_ max between stretch and aerobic groups (*p* = .667, t = 0.44) No significant differences in BDNF between stretch and aerobic groups (*p* = .950, U = 51)	No significant difference in V̇O_2_ max between APOE carriers and non-carriers (relative *p* = .857, *t* = *− *0.18; absolute *p* = .32, *t* = 1.02) Non-carriers in the exercise group showed a significant increase in serum BDNF levels compared to carriers, (*p* = .012, U = 18.0)
Brown et al. (2021) [43] ^a,b,c,d^	99 participants Age = 69.1 54.6% female Cognitively normal	● 50 min ● 2 sessions per week ● 6 months	● Stationary bike 2 groups: moderate (MI) or high intensity (HI) HI: 10 min warm up and cool down, main part of the intervention was 11 1-min intervals at 80% aerobic capacity, broken up by 2 min activity recovery MI: 50–60% aerobic capacity for 50 min Intensity determined by Borg Scale of Perceived Exertion and percentage of peak aerobic power, measured via graded exercise test. Percentage of peak aerobic power for 0–3 months calculated using baseline peak aerobic power output; months 4–6 calculated using peak aerobic power output from mid-intervention fitness test Supervised by an Accredited Exercise Physiologist	Information session on the benefits of exercise for physical health and brain benefits. No exercise instructions	Baseline 3 months (V̇O_2_ peak, peak power, volume of fat, muscle, bone tissue only) 6 months 18 months Global cognitive function ● Digit span ● Cogstate one-back ● Cogstate identification task ● CVLT (learning, short delay recall, long delay recall, and recognition d)` ● BVMT (learning and long delay recall) ● Cogstate Groton Maze recall ● Trails B ● Phonemic fluency ● Flanker ● Set-shifting Attention ● Digit span (forward only) ● Cogstate identification task Episodic memory ● California Verbal Learning Test-II (short delay recall, long delay recall and recognition d`) ● Brief Verbal Memory Test long delay recall ● Groton maze recall Executive function ● Trails B ● Phonemic fluency ● Flanker ● Set-shifting Peak aerobic capacity (V̇O_2_ peak) ● Cycling-based graded exercise test Volume of fat, muscle, and bone tissue (% body fat) ● Dual-energy X-ray absorptiometry scan Peak power ● Cycling-based graded exercise test	85.5% (± 12.4%) in HI and 86.3% (± 9.8%) in MI (no significant difference)	At 6 months, no significant interactions between global cognitive function (*β* = *− *0.04 SE (0.07), attention *β* = 0.00 (0.09), episodic memory, *β* = 0.02 (0.11) or executive function, *β* = 0.01 (0.15) and group assignment, all *p* > .05 Across all timepoints, no significant interactions between global cognitive function (*β* = *− *0.02 SE (0.03), attention *β* = 0.02 (0.04), episodic memory, *β* = *− *0.04 (*− *0.04) or executive function, *β* = *− *0.03 (0.05) and group assignment, all *p* > .05 At 6 months, there was a significant interaction between peak aerobic capacity and group assignment (*β* = 3.67 (0.72), *p* < .001. Follow up tests revealed that HI experienced significant improvements in peak aerobic capacity compared to MI (*β* = 3.92 (no SE given), p < .001) and controls (*β* = 7.36 (no SE given), *p* < .0001). No other findings reported At 18 months, there was no significant interaction between peak aerobic capacity and group assignment (*β* = 0.35 (0.38) At 6 months, there was a significant interaction between % body fat and group assignment (*β* = *− *0.80 (0.37), *p* < .05. HI experienced decreases in % body fat compared to controls (*β* = 1.59, *p* < .005) (No SE given). No other findings reported At 18 months, there was no significant interaction between % body fat and group assignment (*β* = *− *0.09 (0.23) At 6 months, there was a significant interaction between peak aerobic power and group assignment (*β* = 27.33 (4.24) *p* < .001. HI experienced significant improvements in peak power (*β* = 55.62 (no SE given), *p* < .001) compared to controls. No other findings reported At 18 months, there was no significant interaction between peak aerobic power and group assignment (*β* = 3.18 (2.51)	No difference between APOE4 carriers and non-carriers on the cognitive composite scores of global cognitive function, attention, episodic memory, and executive function at 6 or 18 months (statistics not reported) Remaining outcomes not reported by APOE genotype
Cheng et al. (2014a) [44]^c^	110 participants Age = 81.5 Dementia MMSE 10–24 Clinical Dementia Rating > = 0.5	● 60 min ● 3 sessions per week ● 12 weeks	● Tai chi 12 form seated yang style, suitable for frail individuals Intensity not specified Led by research staff	● 60 min ● 3 sessions per week ● 12 weeks Handicraft – participants created shapes by connecting beads	Baseline 3 months 6 months 9 months Primary: ● MMSE Secondary: ● Forward digit sequence ● Backward digit sequence ● Backward digit span ● Forward digit span ● 15-word immediate recall Episodic memory ● 30-min delayed recall Semantic memory ● Categorical verbal fluency (animals, fruit and vegetables – 1 min per category)	Not reported	Statistics were reported assessing interactions across all timepoints Compared to controls, the Tai Chi group significantly improved on the MMSE (*β* = 1.40 95% CI (0.82, 1.97), *p* < .001) at 6 and 9 months (difference between means = 2.3 (95% CI: 0.4, 4.2; d = 0.26, *p* < .05); 3.7 (95% CI: 1.4, 6.0; d = 0.40, *p* < .01) points, respectively; forward digit sequence (*β* = 0.32 (0.03, 0.61), *p* = .030 (though no significant differences at any time point – statistics not reported), and forward digit span (*β* = 0.38 (0.01, 0.76, *p* = .042) at 9 months, difference between means = 0.98, 95% CI: 0.12, 1.84, d = 0.25, *p* < .01 There was no difference between the Tai Chi group and controls on backward digit sequence (*β* = 0.17 (*− *0.08, 0.42, *p* = .184), backward digit span (*β* = 0.14 (*− *0.16, 0.44), *p* = .369),15-word immediate recall, (*β* = 0.10 (*− *0.09, 0.28), *p* = .309); 30-min delayed recall (*β* = 0.09 (*− *0.26, 0.42), *p* = .625); and semantic memory (*β* = 0.66, (*− *0.73, 2.05), *p* = .349 tasks	Statistics were reported across all timepoints; interactions with group assignment were not reported APOE4 carriers had lower scores on the MMSE (*β* = *− *1.90 (95% CI) (*− *3.47, − 0.34), *p* = .017); 15-word verbal immediate recall (*β* = *− *0.34 (*− *0.65, − 0.02), *p* = .036, and 30- min delayed recall (*β* = *− *1.34 (*− *2.5, − 0.18), p = .023, than APOE4 non-carriers There were no significant differences between APOE4 carriers and non-carriers on the forward digit sequence (*β* = *− *0.12 (*− *0.70, 0.42), p = .661, forward digit span (*β* = *− *0.45 (*− *0.97, 0.08), *p* = .095, backward digit sequence (*β* = *− *0.23 (*− *0.69, 0.23), *p* = .318, backward digit span (*β* = *− *0.36 (*− *0.95, 0.23), *p* = .231, and semantic memory (*β* = *− *2.02 (*− *4.21, 0.16), *p* = .070), *p* = .661 tasks
Cheng et al. (2014b) [45]^c^	110 participants Age = 81.5 Dementia MMSE 10–24 Clinical Dementia Rating > = 0.5	● 60 min ● 3 sessions per week ● 12 weeks	● Tai chi 12 form yang style, suitable for frail individuals Intensity not specified Led by research staff	● 60 min ● 3 sessions per week ● 12 weeks Handicraft – participants created shapes by connecting beads	Baseline 3 months 6 months 9 months ● Clinical Dementia Rating sum-of-box (total, cognition, and functioning)	Not reported	Statistics were reported assessing interactions across all timepoints There was no difference between the tai chi and control groups on the CDR sum-of-box total (*β* = *− *0.19, 95% CI (*− *0.62, 0.24), *p* = .390); CDR sum-of-box cognition (*β* = 0.03 (*− *0.21, 0.26), *p* = .813) or CDR sum-of-box functioning (*β* = *− *0.05, (*− *0.29, 0.19), *p* = .664)	Statistics were reported across all time points, interactions were not assessed APOE4 carriers scored significantly differently from non-carriers on the CDR sum-of box total (*β* = 1.63, 95% CI (0.37, 2.89), *p* = .011); and cognition (*β* = 1.17 (0.22, 2.01), *p* = .015), but not functioning (*β* = .404 (*− *0.19, 1.00), *p − *.186)
Eggermont et al. (2009) [46]^c,d^	61 participants Age = 84.6 Moderate dementia (MMSE 11–23)	● 30 min ● 5 sessions per week ● 6 weeks	● Hand movement program, including finger movements, pinching a soft ball, handling a rubber ring Intensity not specified Supervised by recreational therapists or students	● 30 min ● 5 sessions per week ● 6 weeks Stories read by the group leader and conversation	Baseline 6 weeks 12 weeks Memory ● Face recognition (Rivermead Behavioural Memory Test (RBMT)) ● Picture recognition (RMBT) ● Eight Words Test measuring Immediate Recall score and Delayed Recall score and Recognition score Executive function ● Digit Span Forward and Digit Span Backward (Weschler Memory Scale (WMS) – Revised) ● Category fluency ● Stop signal task ● Attention network task Global Cognition ● All memory and executive function tasks Mood ● Geriatric Depression Scale ● Symptom Checklist Anxiety Rest-activity domain Interdaily stability ● Actiwatch activity monitor Intradaily variability ● Actiwatch activity monitor Relative amplitude ● Actiwatch activity monitor	47/61 attended over 80% of the sessions	There were no significant differences between exercise and control groups on the memory domain, F(2, 60) = 0.86, *p* = .43, executive function domain, F(2,60) = 1.11, *p* = .34, cognition domain, F(2,60) = 0.02, *p* = .98, mood domain, F(2,57) = 1.75, *p* = .18, or rest-activity domain, F(2,44) = 0.24, *p* = .79 at any timepoint	There were no significant differences between APOE4 carriers and non-carriers in the intervention and control groups on the domains of memory, F(2, 39) = 0.50, *p* = .61, executive function, F(2, 39) = 0.37, *p* = .70, cognition, F(2, 39) = 0.52, *p* = .60, mood, F(2, 29) = 1.04, *p* = .37 at any timepoint. No analysis was conducted for the rest-activity domain
Eggermont et al. (2009b) [47]^c,d^	97 participants Age = 85.4 81.4% female With moderate dementia (MMSE 11–23)	● 30 min ● 5 sessions per week ● 6 weeks	● Walking at a self-selected speed, with rests as required Intensity not specified Supervised by psychology students	● 30 min ● 5 sessions per week ● 6 weeks Social visits	Baseline 6 weeks 12 weeks Memory ● Face recognition (RBMT) ● Picture recognition (RBMT) ● Delayed recall of the eight words test Executive function ● Digit span backwards ● Category fluency ● Letter fluency Global cognition ● Tests not specified	Not reported	There were no significant differences between the exercise and control groups on the domains of memory, F(2, 94) = 0.15, *p* = .862, executive function, F(2, 95) = 0.37, *p* = .692, or global cognition, F(2, 93) = 0.12, *p* = .887 at any timepoint	There were no significant findings regarding the effect of APOE genotype on the outcomes of memory, F(2, 83) = 0.79, *p* = .460, executive function, F(2, 84) = 0.61, *p* = .547, or global cognition, F(2, 83) = 1.15, *p* = .291 at any timepoint
Galle et al. (2023) [48]^a,b,c,d^	102 participants Age = 70.7 75.5% female MMSE > = 25	● 45 min ● 7 sessions total ● 6 months, with an additional follow-up session at 9 months	● Coaching sessions encouraging daily physical activity: ● Walking ● Cycling ● Housekeeping ● Gardening Low-to-moderate intensity encouraged Supervision during coaching sessions	● 45 min ● 7 sessions total ● 6 months Individually guided whole-body muscle stretching sessions	Baseline 24 weeks 36 weeks Physical activity ● Number of steps by hip-worn pedometer Self-reported physical activity ● Physical activity scale for the elderly Global cognition ● 15 Word Test delayed recall ● Trail Making Test B ● Stroop Colour Word Test Interference ● Letter Fluency Test ● Digit Span backward test Executive function ● Trail Making Test AB-ratio ● Stroop Colour Word Test Interference ● Letter Fluency Test ● Digit Span Test backward Verbal memory ● Delayed recall condition of the 15-Word Test Learning ● Location Learning Test ● 15 Word test Balance ● Short physical performance battery (SPPB) Strength ● Hydraulic JAMAR dynamometer ● Short Physical Performance Battery (SPPB) and handgrip strength (Hydraulic JAMAR dynamomter) Aerobic capacity ● Six-minute walk test Gait speed ● Six-meter walking test Serum concentrations of the cardiovascular risk factor profile ● Total cholesterol, HDL- cholesterol, trigylcerides, insulin, and IGF-1 in serum Psychological well-being ● Rand-36 mental health sub-scale Depressive symptoms ● Centre for Epidemiological Studies Depression Scale Limitations in activities of daily living (ADL) ● Katz-15 Frailty (composite) ● Number of deficits in activities of daily living, social functioning, emotional well-being and self-reported health	Dropout rates under 25%	Statistics were presented at 36 weeks There were significant differences between the exercise and control group on physical activity, *β* = 589.58 (95% CI) (8.78, 1170.40), *p* = .047, *d* = .45, but not self-reported physical activity, *β* = 1.73 (*− *14.14, 17.60), *p* = .83, *d* = .02 There were no significant differences between the exercise and control group on global cognition, *β* = 0.06 (*− *0.31, 0.44), *p* = .76, *d* = .02; executive function, *β* = 0.20 (*− *0.19, 0.59), *p* = .32, *d* = .08; learning, *β* = *− *0.16 (*− *0.46, 0.14), *p* = .29, *d* = .11, or verbal memory, *β* = 0.05 (*− *0.16, 0.25) *p* = .66, *d* = .05 Balance improved significantly in the control group compared to the intervention, *β* = *− *0.76 (*− *1.47, − 0.05), *p* = .04, *d* = .18 There were no significant differences between the intervention and control group on strength, *β* = 0.00 (*− *0.19, 0.20), *p* = .96, *d* = .00; aerobic capacity, *β* = *− *0.63 (*− *19.82, 18.55), *p* = .95, *d* = .01, or gait speed, *β* = *− *0.12 (*− *0.33, 0.09), *p* = .25, *d* = .11 There were no significant differences between intervention and control groups in any of the serum concentrations examined: IGF-1, *β* = 0.21 (*− *0.33, 0.76), *p* = .45, *d* = .05; insulin *β* = 1.44 (*− *3.29, 6.20), *p* = .55, *d* = .04; total cholesterol, *β* = 0.03 (*− *0.09, 0.13), *p* = .69, *d* = .02; HDL- cholesterol, *β* = *− *0.01 (*− *0.05, 0.03), *p* = .62, *d* = .02; and triglycerides, *β* = 0.02 (*− *0.09, 0.13), *p* = .68*, d* = .02 The wellbeing score improved in the control group compared to the intervention group, *β* = *− *2.48 (*− *4.62, − 0.34), *p* = .02, *d* = .21. There was no significant difference between groups on depressive symptoms, *β* = *− *0.86 (*− *2.27, 0.55), *p* = .23, *d* = .14 The number of ADL limitations decreased more in the control group compared to the intervention group, *β* = *− *0.11 (*− *0.21, − 0.01), *p* = .03, *d* = .14 There was no significant difference between the groups on frailty, *β* = 0.00 (*− *0.00, 0.01), *p* = .33, *d* = .00	No interaction of APOE4 carrier status for physical activity, cognition function, and physical fitness (no statistics were reported)
Jensen et al. (2019) [49]^b,c,d^	200 participants Age = 69.8 45.3% female Clinical diagnosis of AD (MMSE > = 20)	● 1 h ● 3 sessions per week ● 16 weeks First 4 weeks were an adaptation to exercise (focusing on strength 2 sessions/week and aerobic exercise 1 session/week) Following 12 weeks were 3 × 10 min aerobic sessions	● Aerobic exercise: ● Treadmill ● Stationary bike ● Cross trainer Moderate-to-high intensity, defined by 70–80% of HRR. Heart rate and perceived exertion were monitored Sessions supervised by an experienced physiotherapist	● Treatment as usual for the first 16 weeks Post exercise intervention, 4 weeks supervised adaptation exercise for 1 h, 3 times /week	Baseline 16 weeks Mental speed and attention ● Symbol Digit Modalities Test Behavioural and psychological symptoms ● Neuropsychiatric inventory Basic mobility ● Timed-up-and-go Gait speed ● 10 m walk test Walking endurance ● Timed 400-m walk test Dual task performance ● Timed 10-m walk test combined with counting backward from 50 V̇O_2_ max ● Estimated based on workload and average heart rate during the last minute of a 6-min cycle test, corrected for age and body weight	83.7% in exercise group		There was an interaction between APOE genotype and group assignment on mental speed and attention, *p* = .0236; such that exercise group APOE4 carriers maintained performance on mental speed and attention; whereas control APOE4 carriers declined There was no interaction between APOE genotype and group assignment for behavioural and psychological symptoms, *p* = .163 There was a significant interaction between APOE genotype and group assignment on basic mobility, *p* = .014; as basic mobility improved significantly more from APOE4 carriers than non-carriers, *p* = .015 There was no significant APOE genotype and group assignment interaction for walking endurance, *p* = .128, and gait speed, *p* = .068 Estimated V̇O_2_ max interaction between group assignment and APOE genotype was non-significant, *p* = .883 Dual task performance was not reported
Karssemeijer et al. (2019) [50]^a,b,c,d^	115 participants Age = 79.2 46.1% female Mild to moderate dementia (MMSE > = 17)	● 20–40 min (progressive increasing by 5 min every 2 weeks until 40 min is reached) ● 3 sessions per week ● 12 weeks	● Stationary bike (with or without additional cognitive training) Intensity was measured by 50–60% of HRR from weeks 1–4; 60–70% HRR weeks 5–9. After 12 weeks 65–75% of HRR should be achieved. Used Borg rate of perceived exertion (RPE) for participants on beta blockers Supervised by trained research assistants	● 30 min ● 3 sessions per week ● 12 weeks Easy relaxation and flexibility exercises	Baseline 6 weeks (executive function tests except letter fluency and all psychomotor speed tests) 12 weeks 24 weeks Executive functioning ● Short form of Trail Making Test Part B ● Abbreviated 5-line Stroop Colour Word Test interference score ● Letter fluency ● Rule Shift Cards Episodic memory ● Location Learning Test-Revised Working memory ● WAIS (Weschler Adult Intelligence Scale)-III Digit Span ● WMS-III Spatial Span Psychomotor speed ● Short form of Trail Making Test part A ● Abbreviated Stroop Colour Word Test parts I and II	81.1% (aerobic group) and 87.3% exergame (aerobic + cognitive stimulation group)	No significant differences between exergame, aerobic group and control group on executive function after 12 weeks, F(2, 115) = 1.01, *p* = .338, or 24 weeks, F(2, 103) = 0.25, *p* = .776; episodic memory after 12 weeks, F(2,115) = 1.72, *p* = *.*184 or 24 weeks F(2,101) = 2.15, *p* = .122 or working memory after 12 weeks F(2, 115) = 1.91, *p* = .153 or 24 weeks F(2, 103) = 2.96, *p* = .056 Significant improvement in psychomotor speed in aerobic and aerobic and cognitive groups compared to controls at 12 weeks, F(2, 115) = 5.77, *p* = .004, η^2^_p_ = .102, and 24 weeks, F(2, 103) = 6.12, *p* = .003 No statistics were presented at 6 weeks	Carrying APOE4 did not influence the relationship between training and cognitive performance (no statistics reported)
Lautenschlager et al. (2008) [51] ^a,b,c,d^	170 participants Age = 68.7 50.6% female Memory problems but no formal diagnosis (> 19 on Telephone Interview for Cognitive Status-Modified; MMSE > 24)	● 50 min ● 3 sessions per week (though some flexibility as aiming for 150 min per week total) ● 24 weeks Participants already achieving 150 min per week were encouraged to carry out an additional 50-min session	● Most frequently recommended walking but other types of exercise were accepted Received a behavioural intervention incorporating a personalised programme workbook Moderate intensity Supervised during interview	Educational material about memory loss, stress management, healthful diet, alcohol consumption, and smoking not physical activity Materials offered to intervention group	Baseline 6 months 12 months 18 months Physical activity ● Community Healthy Activities Program for Seniors survey (minutes per week on all moderate intensity exercise-related activities; minutes per week on all moderate-plus activities and calories expended) ● Pedometer (total steps per week) ● Alzheimer Disease Assessment Scale (ADAS- Cog) ● CDR sum-of-box ● Cognitive Battery of the Consortium to Establish a Registry for Alzheimer Disease total number of words recalled with and without delay (word list immediate recall and word list delayed recall) ● Digit Symbol Coding Test Verbal fluency ● Number of words beginning with F, A, or S in 1 min measured by the Delis-Kaplin Executive Function Battery Frequency and severity of depressive symptoms ● Beck Depression inventory Quality of life ● Medical outcomes 36-item short-form health survey physical and mental composite scores	78.2%	Statistics were presented across all time points (no individual time points were reported) There were no significant differences between groups on the CHAMPS survey (minutes per week on all moderate intensity related activities, *p* = .12; minutes per week on all moderate-plus activities, *p* = .09, and calories expended, *p* = *.*12. The intervention group performed significantly more steps per week than the control group, *p* = .009 The exercise group had significantly higher ADAS-Cog scores. *p* = .04 There were no significant differences between groups on the CDR sum of box, *p* = .05, There was a significant difference on scores between the exercise and control group on the word list delayed recall test, *p* = .02 (larger improvement in the exercise group), but not the word list total immediate recall test, *p* = .48 There were also no significant differences between the exercise and control group on the digit symbol coding test, *p* = .19, verbal fluency test, *p* = .13, Beck Depression Inventory, *p* = .44, physical composite score, *p* = .95, and mental composite score, *p* = .67	APOE4 non-carriers in the intervention group had significantly better ADAS-Cog than other groups combined and APOE4 carriers and non-carriers in the control group, F = 4.59, *p* = .04 and F = 5.01, *p* = .03 respectively. No other significant differences were reported by APOE genotype (no statistics reported)
Legault et al. (2011) [52] ^b,c,d^	73 participants Age = 76.5 50.3% female At risk for cognitive decline, but no MCI (telephone interview for cognitive status > 31)	● Target of 150 min/week, including two 60-min centre-based training sessions per week ● Four months ● 8 visits per month Tailored home-based walking sessions at 1–2 per week for first month Encouraged to increase duration, speed, and frequency, to reach 150 min/week goal	● Primarily walking ● Other endurance activities (e.g. stationary cycling) were used when participants could not walk ● Centre-based sessions were 40 min walking stimulus and 20 min flexibility training Intensity not specified Supervised during centre-based sessions	● Weekly Healthy Aging Education intervention – lectures based on health education. Topics included medication, foot care, travelling and nutrition	Baseline Two months (executive function only) Four months Executive function ● Self-Ordered Pointing Task ● 1-Back and 2-Back tests ● Eriksen flanker task ● Task Switching test ● Trail Making Test Part A and B Episodic memory ● Hopkins Verbal Learning Test immediate recall and delayed recall ● Logical Memory task from Wechsler Memory Scale-III parts I and II Global cognition ● Composite of tests of executive function and episodic memory	Attendance worst in physical activity group (76%)	There were no significant differences between physical activity groups and the control groups on executive function, *p* = .55 episodic memory, *p* = .23, or global cognition, *p* = .66	APOE4 carrier status had no effect on composite cognition, *p* = .70, executive function, *p* = .95, or episodic memory, *p* = .44
Sanders et al. (2020) [53]^c,d^	91 participants Age = 82.3 64.8% female All-cause dementia (MMSE > 10)	● 3 sessions per week ● 24 weeks (12 weeks low intensity (LI), 12 weeks HI	● Walking (outdoors or indoors if weather unsuitable) ● Lower limb strength training programme For walking sessions, intensity determined by heart rate wrist band (percentage of Heart Rate max (HRmax)) and by observer-determined Borg RPE. LI – perceived exertion 9–11 and target HR 57–63% HRmax; HI alternating 4-min peak performance at perceived exertion 15–16 and 83–89% HRmax with 3-min active rest at perceived exertion 13–14 and 71–77% HRmax For strength sessions intensity determined subjectively with observer-determined perceived exertion (LI 9–11; HI 13–16). 0.5 kg ankle weights added for all exercises except toe stands in HI phase One-to-one supervision by a trained research assistant	Flexibility exercises with no weights and recreational activities (board games or social visits depending on preference)	Baseline 12 weeks 24 weeks At 6, 18, and 36 weeks STROOP, leg strength and 6-m walk speed only Endurance ● 6-min walk test Lower body strength and functional mobility ● SPPB Habitual gait speed ● 6-m walking speed test Balance ● FICSIT-4 Quadriso table ● Lower body muscle strength Functional mobility ● Timed up and go Global cognition ● MMSE Psychomotor speed ● Trail Making Test A Verbal memory span ● Digit Span Forward Verbal working memory ● Digit Span Backward Visual memory span ● Visual Memory Span Forward Visual working memory ● Visual Memory Span Backward Attentional processing and inhibitory control ● STROOP (correct word responses; correct colour responses; correct colour-word responses; interference quotient) Executive function ● Phonemic fluency	Mean attendance was ~ 60% for exercise group	After 12, F(1,66) = 2.73, *p* > .05, *d* = 0.18 (95% CI) (− 0.30, 0.65) and 24 weeks, F(1, 66) = 1.36, *p* > .05, *d* = 0.08 (− 0.40, 0.56) there was no significant difference between control and exercise groups on the 6-min walk test There was also no significant difference between the two groups on the SPPB after 12, F(1, 66) = 3.27, *p* > .05, *d* = 0.28 (-0.20, 0.76) and 24 weeks, F(1, 66) = 2.46, *p* > .05 There was a significant difference at 18 weeks for 6-m walking speed, F(1, 66) = 5.12, *p* < .05, and at 24 weeks, F(1, 66) = 12.83, *p* < .001, *d* = 0.41 (− 0.07, 0.90). There were no significant differences reported at 12, F(1, 66) = 1.46, *p* > *.05, d* = 0.04 (− 0.44, 0.52) 6, (statistics not provided), or 36 (statistics not provided), weeks There were no significant differences between the groups on balance at 12, F(1, 66) = 1.45, *p* > .05, *d* = 0.15 (− 0.33, 0.63) or 24 weeks, F(1, 66) = 0.09, *p* > .05, *d* = − 0.15 (− 0.63, 0.33) There were no significant differences between the groups on leg strength at 6 (statistics not provided), 12, F(1,66) = 2.01, *p* > .05, *d* = 0.21 (-0.27, 0.69), 18 (statistics not provided), 24, F(1,66) = 0.39, *p* > .05, *d* = 0.07 (-0.41, 0.55), or 36 weeks (statistics not provided) There were no significant differences between the groups at 12 weeks on the timed-up-and go test, F(1, 66) = 2.35, *p* > .05, *d* = 0.23 (− 0.26, 0.71) or 24 weeks, F(1,66) = 1.43, *p* > .05, *d* = 0.17 (-0.31, 0.66) There were no significant differences between exercise and control groups on MMSE at 12, F(1,66) = 0.11, *p* > .05, *d* = -0.05 (*− *0.53, 0.43) or 24 weeks F(1,66) = 0.04, *p* > .05, *d* = *− *0.04 (*− *0.52, 0.44) There were no significant differences between exercise and control groups on the TMT-A at 12, F(1,66) = 0.51, *p* > .05, *d* = *− *0.03 (*− *0.51, 0.45) or 24 weeks, F(1, 66) = 0.52, *p* > .05, *d* = *− *0.14 (*− *0.62, 0.34) There were no significant differences between exercise and control groups on the digit span forward at 12 weeks, F(1, 66) = 0.96, *p* > .05, *d* = *− *0.03 (*− *0.51, 0.45) or 24 weeks, F(1, 66) = 0.40, *p* > .05, *d* = *− *0.13 (= 0.61, 0.35) There were no significant differences between exercise and control groups on the digit span backwards at 12, F(1, 66) = 0.20, *p* > .05, *d* = *− *0.03 (*− *0.51, 0.45) or 24 weeks, F(1,66) = 0.15, *p* > 05, *d* = 0.10 (*− *0.38, 0.58) There were no significant differences between exercise and control groups on the visual memory span forward at 12 F(1,66) = 2.01, *p* > .05, *d* = 0.04 (*− *0.43, 0.52) or 24 weeks, F(1,66) = 1.20, *p* > 05, *d* = *− *0.05(*− *0.53, 0.43) There were no significant differences between exercise and control groups on the visual memory span backward task at 12 F(1,66) = 0.71, *p* > .05, *d* = 0.14 (*− *0.34, 0.62) or 24 weeks, F(1, 66) = 2.92, *p* > .05, *d* = 0.33 (*− *0.16, 0.81) There were no significant differences between exercise and control groups on the STROOP (correct word responses), at 12 F(1,66) = 0.64, *p* > .05, *d* = 0.07 (*− *0.41, 0.55), 24 weeks, F(1, 66) = 1.35, *p* > .05, *d* = 0.13 (*− *0.35, 0.61) or 6, 18, and 36 (statistics not provided) weeks There were no significant differences between exercise and control groups on the STROOP (correct colour responses) at 12, F(1, 66) = 1.93, *p* > .05, *d* = 0.17 (*− *0.31, 0.65), 24 weeks, F(1, 66) = 0.47, *p* > .05, *d* = 0.03 (*− *0.45, 0.51), or 6, 18, and 36 (statistics not provided) weeks There were no significant differences on the STROOP (colour-word correct responses) at 12, F(1, 66) = 0.45, *p* > .05, *d* = *− *0.24 (*− *0.72, 0.24), 24 weeks F(1, 66) = 0.61, *p* > .05, *d* = *− *0.37 (*− *0.85, 0.12) or 6, 18, and 36 (statistics not provided) weeks There was a significant difference on the STROOP interference quotient at 12 weeks between exercise and control groups at 12 weeks, F(1, 66) = 4.29, *p* = .04, *d* = − 0.49 (*− *0.98, 0.00) (though this did not survive correction for multiple testing), but not at 24 weeks, F(1, 66) = 2.13, *p* > *.05, d* = *− *0.42 (*− *0.90, 0.07), or 6, 18, and 36 (statistics not provided) weeks There were no significant differences between exercise and control groups on the fluency (executive function) task at 12, F(1, 66) = 0.03, *p* > .05, *d* = *− *0.06 (*− *0.54, 0.42) or 24 weeks. F(1, 66) = 2.00, *p* > .05, *d* = 0.13 (*− *0.35, 0.61)	Three-way ANOVA (time/group/APOE genotype) revealed no significant differences on endurance, F(1, 65) = 0.04, *p* > .05, exercise effect size across groups (ES) = 0.15 (95% CI) = (*− *0.49, 0.80), control ES across groups = 0.24 (*− *0.51, 0.98), SPPB, F(1,65) = 0.44, *p* > .05, exercise ES = *− *0.33 (*− *0.98, 0.32), control ES = *− *0.06 (*− *0.80, 0.69), habitual gait speed, F(1, 65) = 1.02, *p* > .05, exercise ES = *− *0.02 (*− *0.67, 0.62), control ES = 0.19 (*− *0.57, 0.94), balance, F(1, 65) = 0.93, exercise ES = *− *0.20 (*− *0.84, 0.45), control ES = 0.21 (*− *0.55, 0.97), leg strength, F(1, 65) = 0.06, *p* > .05, exercise ES = *− *0.25 (*− *0.90, 0.40), control ES = *− *0.06 (*− *0.83, 0.71) or timed up and go, F(1, 65) = 0.29, *p* > .05, exercise ES = *− *0.25 (*− *0.90, 0.40), control ES = *− *0.50 (*− *1.25, 0.25) Three-way ANOVA (time/group/APOE genotype) revealed no significant differences on MMSE, F(1,65) = 3.28, *p* = .075, exercise ES = 0.66 (0.00, 1.32), control ES = *− *0.01 (*− *0.75, 0.73), TMT-A, F(1,65) = 0.69, *p* > .05, exercise ES = 0.19 (*− *0.46, 0.84), control ES = − 0.14 (− 0.88, 0.61), digit span forward, F(1, 65) = 0.21, *p* > .05, exercise ES = − 0.02 (− 0.66, 0.63), control ES = 0.10 (− 0.64, 0.84), digit span backward, F(1, 65) = 0.24, *p* > .05, exercise ES = 0.21 (− 0.43, 0.86), control ES = 0.03 (− 0.71, 0.78), visual memory span forward, F(1, 65) = 0.65, *p* > .05, exercise ES = − 0.06 (− 0.71, 0.58), control ES = 0.32 (− 0.43, 1.06), visual memory span backward, F(1, 65) = 1,70, *p* > .05, exercise ES = − 0.36 (− 1.01, 0.29), control ES = 0.26 (− 0.49, 1.00), STROOP (correct word responses), F(1, 65) = 0.03, *p* > .05, exercise ES = 0.11 (− 0.54, 0.75), control ES = 0.16 (− 0.58, 0.90), STROOP (correct colour responses), F(1, 65) = 1.28, *p* > .05, exercise ES = 0.21 (− 0.44, 0.85), control ES = − 0.19 (− 0.94, 0.55), STROOP (correct colour-word responses), F(1, 65) – 2.56, *p* > .05, exercise ES = 0.73 (0.06, 1.40), control ES = 0.02 (− 0.72, 0.76), STROOP interference quotient, F(1, 65) = 0.87, *p* > .05, exercise ES = 0.66 [0.00, 1.33], control ES = 0.28 (− 0.48, 1.04), or phonemic fluency, F(1, 65) = 0.23, *p* > 05, exercise ES = − 0.11 (− 0.76, 0.53), control ES = − 0.26 (− 1.00, 0.49)
Sindi et al. (2021) [54]^a,b,c,d^	756 participants Age = 69.2 46.6% female Cardiovascular Risk Factors, Aging, and Incidence of Dementia (CAIDE) > 6 and either word list memory task < = 19; word list recall < = 75%; MMSE < = 26	● 30–60 min (progressive) ● 3–8 sessions per week (progressive) ● Two years	Individually tailored, progressive muscle strength training and aerobic exercise programs ● Strength: targets eight muscle groups ● Aerobic: Nordic walking, aqua gym, jogging, gymnastics Intensity not specified Supervised by study physiotherapists	Regular health advice according to established guidelines	Baseline Two years Relative leukocyte telomere length (LTL) ● DNA extracted from peripheral blood Cognition ● Neuropsychological Test Battery (NTB) extended version, total and domain scores for memory, long-term memory, processing speed, and executive function	~ 60% attended > 50% of physical activity sessions [143]	There was no significant difference in LTL maintenance between the intervention and control groups, *β* = 0.007 (95% CI) (− 0.015, 0.030), *p* = .53 Cognitive benefits were more pronounced in participants with LTL maintenance (total NTB score: *β* = 0.127 (− 0.011, 0.264), *p* = .070; executive functioning: *β* = 0.227 (0.057, 0.396), *p* = .009; long-term memory: *β* = 0.257 (0.024, 0.489) *p* = .031 There was no relationship between LTL maintenance and processing speed, *β* = − 0.087 (− 0.268, 0.094), *p* = .347, or memory, *β* = 0.152 (− 0.074, 0.378), *p* = .187	There was better LTL maintenance among APOE4 carriers than non-carriers, *β* = 0.054 (0.007, 0.102) *p* = .026 Relationship with cognition/LTL maintenance/APOE was not investigated
Solomon et al. (2018) [55]^c^	1109 participants Age = 69.3 46.3% female CAIDE > 6 and either word list memory task < = 19; word list recall < = 75%; MMSE < = 26	● 30–60 min (progressive) ● 3–8 sessions per week (progressive) ● Two years	Individually tailored, progressive muscle strength training and aerobic exercise programs ● Strength: targets eight muscle groups ● Aerobic: Nordic walking, aqua gym, jogging, gymnastics Intensity not specified Supervised by study physiotherapists	Regular health advice according to established guidelines	Baseline 12 months 24 months Cognitive performance ● NTB total score Executive function (NTB) ● Category Fluency ● Digit Span ● Concept Shifting Test (condition C) ● Trail Making Test (shifting score B-A) ● Shortened 40-stimulus version of the original Stroop test (interference score 3–2) Processing speed ● Letter Digit Substitution ● Concept Shifting (condition A) ● Stroop (condition 2) Memory ● Visual Paired Associates, immediate and delayed recall ● Logical Memory immediate and delayed recall ● Word List Learning and Delayed recall Abbreviated memory (post hoc) ● 2 associative memory and 2 logical memory tests	~ 60% attended > 50% of physical activity sessions [143]		There was no significant difference on the effect of the intervention by APOE genotype for NTB total score: *β* = 0.023 (95% CI) (− 0.021, 0.067) *p* = .30; executive function, *β* = 0.022 (− 0.034, 0.078), *p* = .44; processing speed, *β* = 0.013 (− 0.047, 0.073), *p* = .68, memory *β* = 0.041 (− 0.031, 0.113), *p* = .27, or abbreviated memory (post hoc), *β* = 0.048 (− 0.030, 0.127), *p* = .22
Solomon et al. (2021) [56]^b,c,d^	1260 participants Age = 69.4 46.7% female CAIDE > 6 and either word list memory task < = 19; word list recall < = 75%; MMSE < = 26	● 30–60 min (progressive) ● 3–8 sessions per week (progressive) ● Two years	Individually tailored, progressive muscle strength training and aerobic exercise programs ● Strength: targets eight muscle groups ● Aerobic: Nordic walking, aqua gym, jogging, gymnastics Intensity not specified Supervised by study physiotherapists	Regular health advice according to established guidelines	Baseline 12 months 2 years ● CAIDE dementia score	~ 60% attended > 50% of physical activity sessions [143]	CAIDE score significantly decreased for intervention group compared to controls, *p* = .013	No significant differences by APOE carrier status, *p* > .032 (no other statistics presented)
Stern et al. (2019) [57] ^a,b,c,d^	132 participants Age = 40.5 70.5% female Cognitively normal	● 40–55 min (10–15 min warm up/cool down and 30–40 min of exercise) ● 4 sessions per week ● 24 weeks	● Aerobic exercise in a Fitness Centre selected from a series of aerobic activities Intensity at weeks 1 and 2 set at 55–65% HR_max_ (determined by a qualifying aerobic capacity test); weeks 3 and 4 65–75% HR_max_; weeks 5–26 75% HR_max_; Contacted by coaches on a weekly basis to monitor progress	Stretch/tone to improve flexibility and core strength targeting all major muscle groups	Baseline 12 weeks (except imaging) 24 weeks Aerobic capacity/ V̇O_2_ max ● Graded exercise test on an electronic-braked cycle ergometer Executive function ● Set switching (letter classification and digit classification) ● CogState Groton Maze Learning Test Episodic memory ● Modified Rey Auditory Verbal Learning Test ● CogState Continuous paired associate learning Processing speed ● Digit symbol (WAIS)-III digit symbol subtest) ● Cogstate Groton Maze Chase Test ● Cogstate Identification Task Language ● Controlled Oral Word Association Test ● Animal naming Attention ● The 2 and 7 test Working memory ● WAIS-III letter-number sequencing ● Cogstate N-back Everyday function ● Timed instrumental ADL tasks ● BMI Cortical thickness ● T1-weighted magnetisation-prepared rapid gradient echo scan	33.33% attrition rate for exercise group	V̇O_2_ max significantly increased in the intervention group at 12 (*β* = 3.139 (95% CI) (1.476, 4.802), *p* < .001 and 24 weeks (*β* = 2.718 (0.931, 4.505), *p* = .003 compared to controls There was no difference between groups on executive function at 12 weeks, *β* = 0.166 (− 0.048, 0.379) *p* = .128, but the intervention group scored better at 24 weeks, *β* = 0.237 (0.014, 0.461), *p* = .038 Episodic memory was not significantly different between the groups at 12, *β* = 0.019 (− 0.193, 0.231), *p* = .863, or 24 weeks, *β* = − 0.037 (− 0.259, 0.185) *p* = .741 weeks Processing speed was not significantly different between the groups at 12, *β* = 0.068 (− 0.099, 0.235), *p* = .425, or 24 weeks, *β* = 0.007 (− 0.168, 0.181) *p* = .941 Language was not significantly different between the groups at 12, *β* = 0.029 (− 0.165, 0.223), *p* = .771, or 24 weeks, *β* = − 0.062 (− 0.266, 0.141) *p* = .546 Attention was not significantly different between the groups at 12, *β* = − 0.105 (− 0.570, 0.36) *p* = .657, or 24, *β* = − 0.233 (− 0.721, 0.254), *p* = .347, weeks Working memory was not significantly different between the groups at 12, *β* = − 0.214 (− 0.496, 0.068), *p* = .136 or 24, *β* = 0.027 (− 0.267, 0.322), *p* = .856 weeks There was no significant different between the groups on everyday function at 24 weeks, *β* = − 0.368 (− 1.215, 0.479), *p* = .390. (12 weeks not reported) BMI was not significantly different between the groups at 12 weeks, *β* = − 0.374 (− 0.816, 0.068), *p* = .096, but decreased more in the intervention group after 24 weeks, *β* = − 0.596 (− 1.062, − 0.129), *p* = .013 The intervention was associated with significantly increased cortical thickness in left caudal middle frontal cortex Brodmann area (no statistics given)	APOE4 carriers showed less improvement in executive function with aerobic exercise than non-carriers, *β* = 0.523 (0.038, 0.988), *p* = .035. No difference in other cognitive areas (statistics not presented)
Stonnington et al. (2020) [58]^a,b,c,d^	53 participants Age = 63.4 100% female Cognitively normal (MMSE > 28)	● 60 min (first 5 min warm up and last 5 min cool down) ● At least 40 sessions over 6 months	● Zumba: dance and aerobic movements, including samba, salsa, merengue, mamo, hip-hop, squats, or lunges, to music Supervised by Zumba instructors	Maintenance of habitual exercise	Baseline 3 months 6 months Visuospatial working memory, error monitoring, information processing speed, short-term delayed recall for a complex hidden maze ● Groton maze learning test Visual recognition memory and learning ● One-card learning Working memory, attention ● Two-back test Impulsivity, inhibition ● Set-shifting Visual learning and memory ● Continuous paired associate learning Executive functioning ● Delis-Kaplan Executive Functioning System Colour-Word Interference Test ● Delis-Kaplan Executive Functioning System Sorting Test Visual attention, task-switching ● Trail-making Test parts A and B Verbal memory ● Rey’s Auditory Verbal Learning Test long-term memory score Quality of life ● Short Form Health Survey (SF-36) Kcal/wk total exercise and kcal/wk moderate intensity ● Community Healthy Activities Model Program for Seniors BMI Aerobic fitness ● 6-min walk test (baseline only) Enjoyment of Zumba, ability to follow dance moves, and the feeling that it was a good work-out (intervention group only – weekly) ● Ordinal scale	Median number of sessions per week attended was 2 in intervention group	(Statistics reported after 6 months alone) There was no significant difference between exercise and control groups at 6 months in Groton Maze Learning Test total errors (delta = 2.0 (effect size) (0.07); 95% CI (− 7.1, 12), *p* = .59 There were no significant differences between groups on the one-card learning accuracy score, delta = 0.01 (0.13) (− 0.03, 0.05), *p* = .61 There were no significant differences between groups on the two-back accuracy score, delta = − 0.02 (0.16) (− 0.07, 0.04), *p* = .61 There were no significant differences between groups on the set-shifting task accuracy, delta = 0.05 (0.47) (− 0.01, 0.11), *p* = .08 There were no significant differences between groups on the continuous paired associate learning task accuracy score, delta = − 0.03 (0.16) (− 0.14, 0.08), *p* = .61 There were no significant differences between the groups on the Delis-Kaplan Executive Functioning System Colour-Word Interference test, delta = − 3.3 (effect size = 0.26) (− 8.6, 2.1), *p* = .23 There were no significant differences between the groups on the Delis-Kaplan Executive Functioning System Sorting Test, delta = 2.0 (0.72) (− 0.7, 4.7), *p* = .22 There were no significant differences between the groups on the Trail Making Test – B, delta = − 2.0 (0.07) (− 11, 7.1), *p* = .67. (TMT-A not reported) There were no significant differences between groups on the Rey’s Auditory Visual Learning Test, delta = 0.9 (0.29) (− 0.3, 2.1), *p* = .15 There were no significant differences between groups on the Short-Form Health Survey Physical Composite Score, delta = 2.1 (0.57) (− 0.8, 5.0), *p* = .15 There were no significant differences between groups on the Short-Form Health Survey Mental Composite Score, delta = 1.0 (0.16) (− 1.9, 3.9), *p* = .50 There were no significant differences at 6 months on total exercise (kcal/week) (delta: 1.8 (95% CI) (− 1.5, 5.2) *p* = .28; moderate exercise (kcal/week) (delta = 1.7 (− 0.9, 4.2) *p* = .19; amount of exercise per week (delta: 0.81 (− 0.62, 2.24) *p* = .26 No significant differences in BMI (statistics not reported)	No APOE carrier status interaction (no statistics reported; except for post hoc tests not reported here)
Vidoni et al. (2021) [59]^a.b.c.d^	117 participants Age = 72.9 66% female No cognitive impairment but elevated or subthreshold cerebral amyloid levels	● No more than 50 min ● 3–5 sessions per week ● 52 weeks Started with an aim of 60 total minutes of exercise in week 1 which increased by 21 min/week until 150 min/week of aerobic exercise was achieved	● Aerobic exercise (primarily walking) but allowed other forms to avoid boredom Intensity set as target heart rate zone (40–55% of HRR to start, then increased by 10% of HRR every 3 months) Supervised by certified personal trainers for the first 6 weeks of exercise and at least once/week after this time	Education control, provided with public health information about exercise and on completion of the study received membership to a community gym	Baseline Week 26 (except V̇O_2_ max or MRI) Week 52 Global amyloid burden ● 18F – AV45 PET scan standard uptake value ratio Hippocampal volume & total grey matter (whole brain) volume ● Siemans 3.0 Tesla Skyra scanner Executive function ● Verbal fluency (sum of animals and vegetables) ● Trail making test B ● Digit symbol substitution test ● Interference portion of Stroop Verbal memory ● Immediate and delayed portions of the logical memory test ● Sum of free recall trials of the selective reminding test Visuospatial domain ● Block Design ● Space relations ● Paper folding test ● Hidden pictures ● Identical pictures Cardiorespiratory fitness testing: V̇O_2_ max ● Graded maximal exercise test	Participants completed an average of 84.6% minutes of the exercise sessions	There were no significant differences between global amyloid burden between the groups after 52 weeks, *p* = *.*93 There were no significant differences between the groups after 52 weeks for whole brain volume, *p* = .12, or hippocampal volume, *p* = .42 There were also no differences between the groups on the executive function composite at 26 or 52 weeks (treatment by time interaction), *p* = .83; verbal memory composite, *p* = .69, or visuospatial composite, *p* = .30 There were significant differences between the two groups after 52 weeks for V̇O_2_ max, *p* = .01	No differences by APOE carrier status (data not shown)
Yu et al. (2022) [60]^b,c^	26 participants Age = 77.6 35.6% female Mild-to-moderate AD (MMSE 15–26; Clinical dementia rating 0.5–2)	● 20–50 min ● 3 sessions per week ● 6 months	● Stationary cycling Intensity gradually increased from 50–55% HRR or rate of perceived exertion 9–11 for 20–30 min to 70–75% HRR or rate of perceived exertion 12–14. Increases in intensity were alternated with session duration (5-min increases and 5% intensity/1 point perceived exertion increases) Supervised	● Matched to exercise group Seated movements at static stretching at low intensity (< 20% HRR or < 9 rate of perceived exertion)	Baseline 3 months 6 months Feasibility ● Recruitment rate, retention rate, and blood collection rate Plasma Ab_42/40_ ratio, plasma t-tau, and p-tau181 ● Simoa assays	84.8% of exercise sessions were attended	Recruitment rate was 76.5%; retention rate was 100% at 3 months and 96.2% at 6 months. The rate of blood collection was 88.5% at 3 months and 96.2% at 6 months For plasma Ab_42/40,_ ratio there was no significant difference between the groups at 3 (*β* = − 0.003 (95% CI) (− 0.006, 0.001), *p* > .05, or 6 months ( *β* = − 0.002 (− 0.006, 0.001), *p* > .05 For plasma t-tau, there were no significant differences between the groups at 3 (*β* = 0.032 (− 0.137, 0.201), *p* > .05, or 6 months (*β* = − 0.040 (− 0.206, 0.127), *p* > .05 There was also no significant difference between the two groups for p-tau181 at 3, (*β* = 0.218 (− 0.561, 0.996), *p* > .05) or 6 (*β* = − 0.210 (− 0.988, 0.567), *p* > .05, months	Compared to the APOE e3/e4 genotype, there was no difference on the e2/e3 genotype for plasma Ab_42/40_ ratio, *β* = − 0.007, 95% CI (− 0.028, 0.013), *p* > .05, or e2/e4 genotype, *β* = − 0.007 (− 0.018, 0.003), *p* > .05, or e4/e4 genotype, *β* = − 0.002 (− 0.012, 0.008) Compared to the APOE e3/e4 genotype, there was no difference on plasma t-tau levels for e2/e3, *β* = 0.179 (− 0.209, 0.567), *p* > .05 or e4/e4, *β* = − 0.046 (− 0.239, 0.147), *p* > .05; but APOE e2/e4 had a significantly lower plasma t-tau level than APOE e3/e4 carriers, *β* = − 0.234 (− 0.425, − 0.043), *p* < .05 There was no significant difference between the e3/e4 genotype and the APOE e2/e3 genotype on p-tau181 levels, *β* = 1.827 (− 0.794, 4.448), *p* > .05, or e2/e4 levels ( *β* = − 0.811 (− 2.079, 0.457), or e4/e4 levels (*β* = − 0.228 (− 1.516, 1.060), *p* > .05

Efforts were made to report all statistical findings from intention-to-treat analyses that were presented in the included articles. Post hoc statistical tests for unplanned analyses are not reported. Many studies were missing key statistical information for one or more of their outcomes (see key below), and selectively reported findings for significant results

a) No *p*-value provided (only significant.vs non-significant)

b) No test-statistics provided

c) No estimated effect size provided

d) No confidence interval provided

MMSE [62]; Modified Bruce Protocol [63]; Borg Scale Perceived Exertion (RPE) [64]; Clinical Dementia Rating (CDR) [65]; forward digit sequence [66]; forward digit span [66]; backward digit sequence [66]; backward digit span [66]; categorical verbal fluency [67]; Rivermead Behavioural Memory Test (RBMT) face recognition [68]; picture recognition [68]; Eight words test [69]; Weschler Memory Scale (WMS)-Revised digit span [70]; category fluency [71]; attention network test [72]; geriatric depression scale [73]; Symptom Checklist Anxiety [74]; Physical Activity Scale for the Elderly [75]; 15 Word Test delayed recall [76]; Stroop Color Word Test Interference score [77]; Letter Fluency Test [78]; Digit Span backward test [70, 79]; Location Learning Test [80, 81]; Short Physical Performance Battery (SPPB) [82]; Six Minute Walk Test [83]; Rand-36 [84]; Centre for Epidemiological Studies Depression Scale [85, 86]; Katz-15 [87]; Symbol Digit Modalities Test [88]; Neuropsychiatric Inventory [89]; Timed-Up-and-Go [90];Timed 10-m walk test [91]; timed 400-m walk test [92]; dual task performance [91]; V̇O_2_ max [93–96]; Short form Trail Making Test part B [97–100]; abbreviated 5-line Stroop Colour Word Test interference score [101], Letter Fluency [102, 103]; Rule Shift Cards Test [104]; Location Learning Test- Revised [105]; Wechsler Memory Scale-III Spatial Span [79]; abbreviated Stroop Colour Word Tests parts I and II [101]; Community Healthy Activities Program for Seniors [106]; Alzheimer Disease Assessment Scale (ADAS-Cog) [107]; Digit Symbol Coding Test [108]; Delis-Kaplin Executive Function Battery [109]; Cambridge Contextual Reading Test [110]; Beck Depression Inventory [111]; Medical Outcomes 36-Item Short-Form Health Survey [112–114]; Self-Ordered pointing task [115]; 1-back test [116]; 2-back test [117]; Eriksen flanker task [118]; task switching test [119]; Hopkins Verbal Learning Test [120], Wechsler Memory Scale-III [79]; 6 min walk test [83]; Short Physical Performance Battery (SPPB) [82]; FICSIT-4 [121]; Quadriso tester [122]; Span Forward [97] Digit Span Backward [97]; Visual Memory Span Forward [97]; Visual Memory Span Backward [97]; STROOP [123]; phonemic fluency [124]; Neuropsychological Test Battery (NTB) Extended [125]; Cardiovascular Risk Factors, Aging, and Incidence of Dementia (CAIDE) [126]; Set switching [127, 128]; Groton Maze Learning Test (CogState) [129]; Modified Rey Auditory Verbal Learning Test [130, 131]; Continuous paired associated learning (CogState) [129]; Digit symbol (Wechsler Adult Intelligence Scale [WAIS]-III digit symbol subtest [108]; Groton Maze Chase Test (CogState) [129]; Identification task (CogState) [129]; Controlled Oral Word Association Test [132]; Animal naming [133]; the 2 and 7 test [134]; WAIS-III letter-number sequencing [108]; N-back (CogState) [129]; Timed instrumental activities of daily living tasks (TIADL) [135]; Groton Maze Learning Test [136]; One- card learning task (OCL) [129]; Delis-Kaplan Executive Functioning System (DKEFS) Color-Word Interference Test [109]; Trail-making Test parts A and B [71]; Rey’s Auditory Verbal Learning Test [137]; Short-Form Health Survey (SF-36) [114]; Community Healthy Activities Model Program for Seniors physical activity questionnaire for older adults [106]; 6-min walk test [138, 139]; verbal fluency (sum of animals and vegetables [133]; Trail making Test B [140]; Digit Symbol Subsitution test [79]; interference portion of the Stroop test [77]; Logical memory Test [79]; sum of free recall trials of the Selective Reminding Test [141]; Block Design [79]; space relations [142]; paper folding test [142]; hidden pictures [142]; identical pictures [142]

Participants characteristics, exercise schedule and mode, control parameters, outcome measures, adherence, and findings were extracted from the 19 studies deemed suitable for inclusion in the systematic review

The mean age of participants ranged from 40.45 (13.42) [57] to 85.4 (SD not reported) [47]; with an average age of 72.29 (10.55) years old. The cognitive status of participants also varied, ranging from cognitively healthy (5/19), to at elevated risk for dementia or mild cognitive impairment (MCI) (4/19), to individuals with MCI or subjective memory problems (2/19), and living with dementia (6/19), or specifically AD (2/19).

Out of 19 included studies, 8 had between approximately 50% (40–60%) female participants, 7 had over 60% female participants, and 3 did not report this characteristic. The only exception was the study by Yu et al. [60] which consisted of 35.6% (9/26) female participants. The majority (14/19) of included studies did not report the ethnicity of participants. Of those that did, participants were predominately from White backgrounds (4/19); with the exception being the 100% African American cohort included in the study by Allard et al. [42].

#### Intervention

The duration, frequency, and intensity of exercise interventions varied between studies. Sessions ranged from 20 to 60 min in length, on a schedule ranging from one session every 3 to 5 weeks up to eight sessions per week. As defined by intensity values in each paper, studies focused on moderate- to high-intensity exercise; with cycling (6/19) and walking or jogging (8/19), the most common forms of exercise utilised. Despite this focus, exercise intensity varied between studies; low-intensity exercise included hand movement exercises and tai chi [44–46] and high-intensity exercise typically focused on cycling [49].

The nature of control groups formed four categories: ‘active’ (6/19) groups performed stretching and toning or flexibility exercise, typically on the same schedule as the exercise group; ‘education’ (7/19) groups received information about healthy lifestyles; ‘social’ (5/19) groups received visits from research staff or took part in group activities; and ‘treatment as usual’ (2/19) groups maintained their current activity levels. One control group [57] was offered both flexibility exercises and social activities.

All studies provided some level of human supervision during the exercise sessions, which was usually conducted by research assistants and physiotherapists. Supervision was often reduced throughout the course of the interventions as participants became more confident with the required exercise.

Adherence rates were equal to other exercise-based RCTs. Martin et al. [144] report that, on average, older adults complete 78% of exercise sessions in RCTs. Here, 6/19 studies reported the proportion of exercise sessions completed, with an average of 82% (SD: 3.80); however, this does not account for the large number of studies which do not provide adherence statistics on the number of sessions attended in the exercise group.

#### Outcomes

All studies measured outcomes immediately after the exercise intervention was completed. However, the time to last follow-up post exercise intervention varied from immediate on completion of the exercise intervention, to 12 months after the intervention [43, 51]. From baseline, the mean length of time until final assessment was 10.61 (SD: 7.33) months. Cognitive outcomes were measured more commonly than physical outcomes. The most commonly assessed cognitive outcome was executive function (11/19), and V̇O_2_ max (5/19) was the most commonly recorded physical outcome. The number of outcome measures recorded varied across included articles from one (e.g. [56]) to sixteen [46] (median: five). As a result of the included articles measuring multiple outcomes, over 50 different outcomes in total have been measured by studies in this review.

Of the studies included in the systematic review, none had a primary aim to examine outcomes by APOE genotype, so these studies were likely to have been underpowered to detect differences between APOE genotypes if they were present (findings summarised in Table 2; Additional file 2). Generally, studies were of very low- to moderate-quality evidence, and findings were variable as some outcomes were reported as statistically different in one study but may be reported as not significantly different in another. Effect sizes and confidence intervals were not often reported which limited interpretation of the findings; and where they were reported, effect sizes were typically small to medium, and confidence intervals were often wide highlighting the imprecise nature of the results found.

Although some individual studies did report significant findings suggesting there may be a benefit on some outcomes for APOE4 carriers or non-carriers; findings were not typically replicated across studies. Furthermore, although some studies showed significant results; the GRADE assessment (see below) found that many of the outcomes were downgraded on imprecision and risk of bias, which raises questions about the reliability of the findings.

#### Risk of bias

Analysis of risk of bias by ROB2 [38] demonstrated that most (14/19) studies had some concerns of a risk of bias. The remainder had a low risk of bias except [58] which presented a high risk of bias (Table 3; see additional file 3 for complete ROB2 assessment). Bias was primarily identified in domains examining the randomisation process, deviations from the intended intervention and selection of the reported result.

**Table 3:**
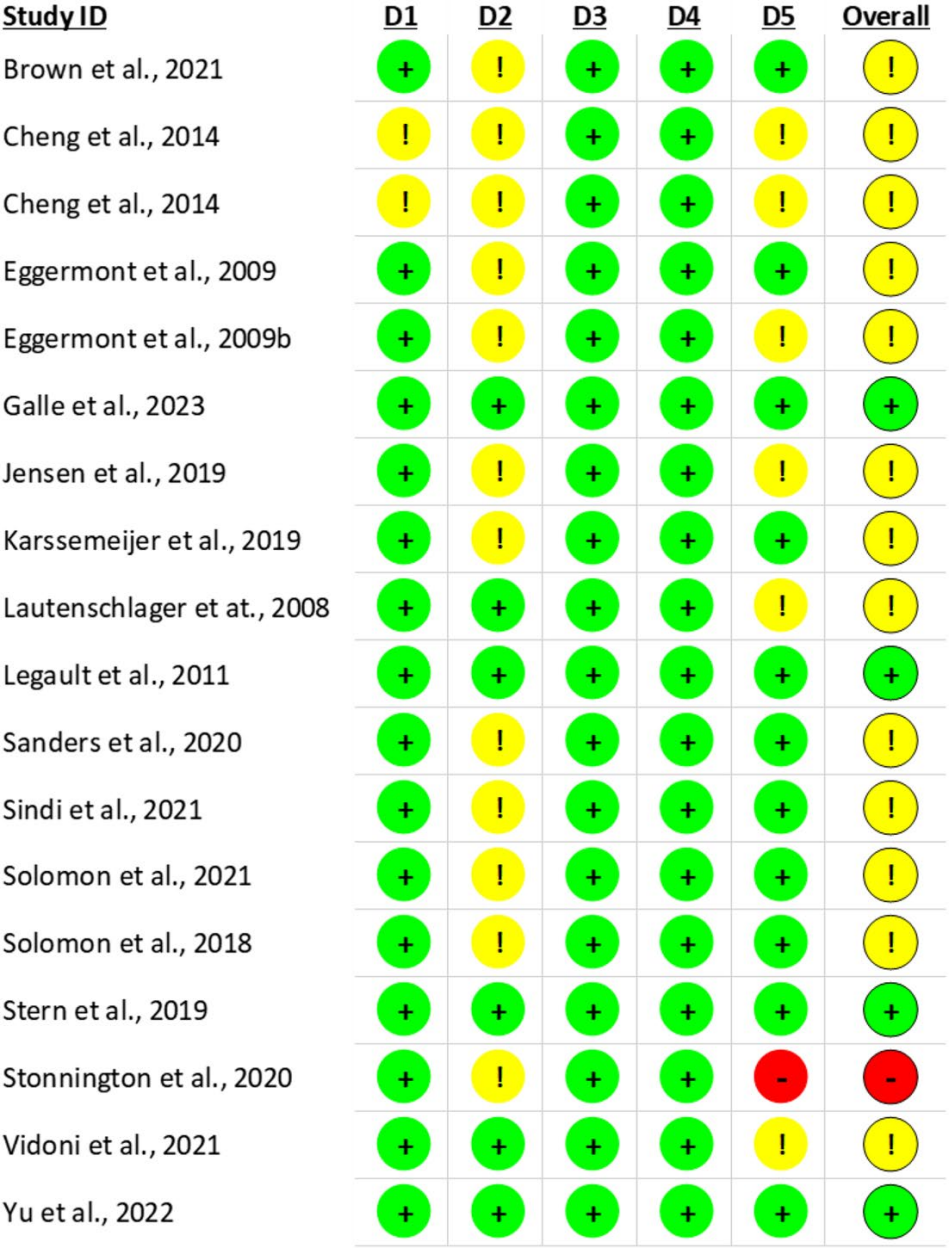
Scores on the ROB2

This is the results of the assessment of risk of bias for the included studies using the ROB2 tool.—indicates high risk of bias; ! indicates some concerns of risk of bias; and + indicates low risk of bias. D1 = randomisation; D2 = deviations from the intended intervention; D3 = missing outcome data; D4 = measurement of the outcome; D5 = selection of reported results

#### Quality assessment

Some key areas for quality improvement were identified by the CASP [36] assessments. A lack of diversity in participant groups in the included articles was identified alongside a lack of reporting of power calculations and effect sizes. Furthermore, APOE carriers were not often equally distributed between exercise and control groups. Typically, areas of good quality were addressing a focused research topic, treating groups equally, and applicability of results to the population at risk.

Total TESTEX [37] scores ranged from 5 to 12/15 (M = 8.68; SD = 1.87) (Table 4).

**Table 4:** Scores on TESTEX

Author	Eligibility criteria specified (1)	Randomisation specified (1)	Allocation concealment (1)	Groups similar at baseline (1)	Blinding of assessor (1)		Outcome measures assessed in 85% patients (3)	Intention to treat analysis (1)	Between group statistical comparisons reported (2)	Point measures and measures of variability (1)	Activity monitoring in control groups (1)	Relative exercise intensity remains constant (1)	Exercise volume characteristics and energy expenditure (1)	Total
Allard (2017) [42]	1	0	1	0		1	0	0	2	1	0	0	1	7
Brown (2021) [42]	1	1	1	0		0	2	1	2	1	0	1	1	11
Cheng (2014a) [43]	1	1	0	1		0	0	0	2	0	0	0	0	5
Cheng (2014b) [44]	1	1	1	1		0	0	0	1	1	0	0	0	6
Eggermont (2009a) [45]	1	1	1	1		1	0	0	2	0	0	0	0	7
Eggermont (2009b) [46]	1	1	1	0		1	0	0	2	1	0	0	0	7
Galle (2023) [47]	1	1	1	0		0	0	1	2	1	1	0	0	8
Jensen (2019) [48]	1	1	1	1		1	1	1	2	0	0	0	1	10
Karssemeijer (2019) [49]	1	1	1	0		1	1	1	2	1	0	0	1	10
Lautenschlager (2008) [50]	1	1	1	0		1	2	1	2	1	1	0	0	11
Legault (2011) [51]	1	0	1	1		0	1	1	2	0	0	0	0	7
Sanders (2020) [52]	1	1	1	0		1	1	0	2	1	0	0	1	9
Sindi (2021) [53]	1	1	1	1		1	1	0	1	0	0	1	0	8
Solomon (2021) [54]	1	1	1	1		1	2	1	1	1	0	1	0	11
Solomon (2018) [55]	1	1	1	0		1	1	0	2	1	0	1	0	9
Stern (2019) [56]	1	1	1	1		1	1	1	2	0	0	0	1	10
Stonnington (2020) [57]	1	1	1	1		1	0	0	2	0	1	0	0	8
Vidoni (2021) [58]	1	1	1	1		1	1	1	2	1	1	0	1	12
Yu (2022) [59]	1	1	1	0		1	0	1	2	1	0	0	1	9

This is the result of ranking on the TESTEX. The numbers indicate how many points were awarded for each section

Marks were often lost on the study reporting section, for the reporting of adherence outcomes and adverse events; monitoring and reporting the activity levels of the control group; progressively increasing the intensity of the exercise intervention and complete reporting of the exercise protocol. Marks were typically earned on the study quality section, for having a clearly defined research question and eligibility criteria; having similar groups at baseline; and blinded outcome assessors.

#### Meta-analysis

As a result of lack of data availability (descriptive statistics were usually not presented disaggregated by APOE genotype, requests for data were rarely answered), only outcomes relating to physical fitness or physical function of V̇O_2_ max, gait speed, walking endurance, and mobility were able to be synthesised. Analyses compared exercise and control conditions, with data from APOE4 non-carriers and APOE4 carriers entered in separate subgroups. Only three studies provided available data for these analyses, which included a total of 288 participants; as such further disaggregation of the data by sex and ethnicity was not feasible. Participants were a mix of cognitively normal (1/3) and living with dementia (1/3) and AD (1/3).

All tests of heterogeneity for the complete outcome (not sub-groups) were non-significant, *p* > 0.05. However, as a result of the small number of studies eligible for inclusion in the meta-analysis, the tests for heterogeneity have limited power, so interpretation of the differences between studies using this test was limited. To be cautious, random effects models were used in the meta-analysis to account for heterogeneity [38].

**Fig. 2:**
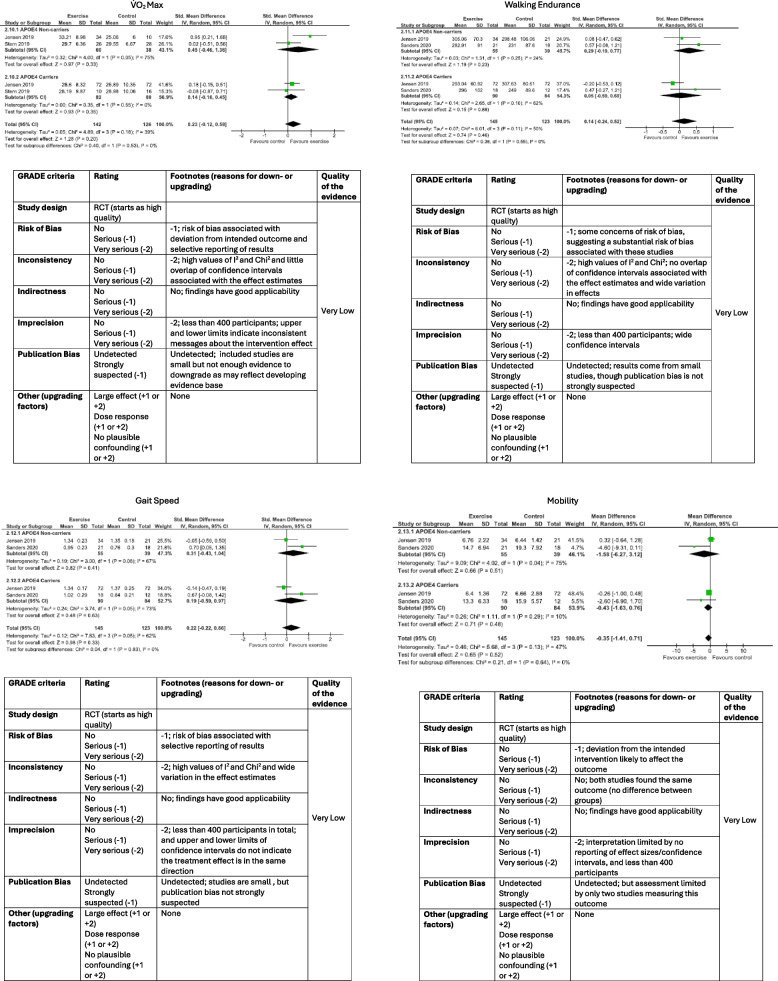
Forest plots and GRADE summary tables examining physical outcomes by APOE genotype. Legend: The forest plots compare scores from exercise and control conditions, separated into APOE4 carrier and APOE4 non-carrier subgroups for the outcomes of V̇O_2_ max, walking endurance, gait speed, and mobility. Tests for heterogeneity are given as well as the estimated effect for each subgroup, and the overall effect for each outcome. In the corresponding forest plot, the size of the box corresponds to the weight of each study, the position relates to the difference between the exercise group and control group. The lines represent the 95% confidence interval of each effect. The position of the diamond represents the overall effect size. The relevant GRADE summary tables are displayed below each forest plot

V̇O_2_ max, walking endurance, gait speed, and mobility showed non-significant differences between exercise and control conditions for both APOE4 carriers and APOE4 non-carriers, all *p* > 0.05, suggesting that there was no evidence of differences between APOE4 carriers and non-carriers on these outcomes (Fig. 2).

However, all outcomes were rated as very low quality by GRADE assessment (Fig. 2), meaning that these findings may not reflect the ‘true’ size of difference between the two APOE4 groups. High levels of uncertainty in the estimated effects means the magnitude and direction of any difference between the two groups remains unclear. The confidence intervals of the estimates of treatment effect typically extended in both directions indicating that it was unclear whether exercise resulted in a benefit or harm on the outcome.

#### Publication bias

The ROB-ME revealed low concerns of risk of bias due to missing evidence for each of the four meta-analyses (see additional file 4).

#### Sensitivity analysis

Sensitivity analyses were conducted for the meta-analysis whereby studies with some concerns of risk of bias or high risk of bias as identified in the ROB2 were removed from the meta-analysis. This was not possible for the outcomes of walking endurance, gait speed, and mobility as both studies included in these analyses were identified as having either some concerns or a high risk of bias. For the V̇O_2_ max outcome, removal of the study with some concerns of bias did not have a large effect on the results (APOE4 non-carriers standardised mean difference = 0.02 [− 0.51, 0.56], *Z* = 0.09, *p* = 0.93; APOE4 carriers standardised mean difference = − 0.08 [− 0.87, 0.71], *Z* = 0.19, *p* = 0.85, overall effect, *Z* = 0.03, *p* = 0.97), though only one study was left remaining in the meta-analysis, limiting interpretation of this finding.

#### GRADE

GRADE [41] assessment was conducted for each of the outcomes assessed in the meta-analysis and systematic review. The quality of evidence for outcomes in the meta-analysis were rated as very low. Primary reasons for downgrading were serious or very serious issues with inconsistency (little overlap of confidence intervals) and imprecision (less than 400 total participants and large confidence intervals extending in both directions), alongside risk of bias (issues with selective reporting of results and deviation from the intended intervention) (see Fig. 2).

## Discussion

This systematic review and meta-analysis was conducted to assess whether carrying APOE4 impacts the effectiveness of exercise (assessed in RCTs) on physical and cognitive health outcomes, and to examine the quality of the methodology used in these studies. There were 19 articles identified as suitable for inclusion in the systematic review, and 3 for inclusion in the meta-analysis. There was no evidence of differences on physiological outcomes between APOE4 carriers and non-carriers. However, the main objective of assessing the impact of APOE4 genotype on exercise trials could not be fully addressed by the available studies due to a lack of data. Individual studies did not focus on assessing the outcomes by APOE genotype; participants were rarely stratified by APOE genotype, and no studies had a primary outcome to assess the effect of APOE genotype in the RCT.

The results of the systematic review demonstrate that research in this area is varied. A wide range of participants, exercise and control group schedules, and outcome measures were studied. As a result, findings are also mixed which makes it difficult to understand what exercise is optimal to provide the most benefit (mode, intensity, duration, frequency, etc.). The mixed findings may be the result of the heterogenous study characteristics, or may arise from the very low to moderate quality of the available evidence.

Despite many reported findings suggesting that benefits of exercise to APOE4 carriers and non-carriers exist; results were not reliably reproduced, and, where reported, average effect sizes were typically small to medium demonstrating that the impact of findings in the real world may be quite limited. The conclusions that can be made about the effectiveness of exercise intervention on APOE4 carriers and non-carriers are also limited. Although many individual studies reported improvements in participants regardless of APOE4 carrier status, the quality of available evidence, when synthesised, undermined many of these conclusions.

Previous reviews in this area have demonstrated that both APOE4 carriers and non-carriers can benefit from exercise intervention. de Frutos-Lucas et al. [30] reported that both APOE4 carriers and non-carriers benefitted from physical activity in observational studies; though in studies with larger cohorts, APOE4 carriers showed a greater effect of intervention, concluding that findings were inconclusive overall. Furthermore, a review by Cancela-Carral et al. [31] demonstrated that higher intensity exercise and exercise performed for more than 50 sessions predominantly benefitted APOE4 non-carriers. This finding was not replicated herein due to a lack of data availability to be able to perform these comparisons.

The range of effect sizes observed in the meta-analysis resulted in no evidence of significant differences on measures of physical function between APOE4 carriers and non-carriers, though only four outcomes could be assessed due to a lack of available data. The findings suggest that exercise programs may have clinical importance for APOE4 carriers and non-carriers as both may benefit from exercise intervention. However, all outcomes were rated as very low quality by GRADE assessment, demonstrating that the certainty of the evidence is very low. Therefore, conclusions about the effectiveness of exercise intervention on APOE4 carriers and non-carriers cannot be made from this synthesis.

The studies reviewed show that RCTs with an exercise intervention can provide varied benefits for a range of individuals. However, some factors make interpretation of these studies challenging. Firstly, the range of outcome measures used across the included articles make it difficult to compare results. The recent development of a core outcome set for physical activity-based clinical trials for people with dementia [149] may help to reduce variability in future studies, though was not adopted by any of the studies included in this review. Additionally, for most outcomes, it is unclear what constitutes a clinically meaningful difference following intervention. Therefore, findings are often interpreted in terms of their statistical significance alone, rather than whether an individual experiences a tangible improvement following intervention. Guidelines on detecting a clinically meaningful difference for the CDR [65], MMSE [62], and ADAS-Cog [107] may improve use of these scales, and interpretation of results [150]. Furthermore, many analyses of outcome measures by APOE carrier status were secondary analyses. Often, participants were not stratified by APOE carrier status, so group sizes were uneven. This makes it difficult to determine if results reflect the ‘true’ state of the literature.

The quality analysis revealed that there are several areas in which RCTs in this area consistently lack quality. Findings from the CASP [36] demonstrated that the majority of included articles did not report ethnicity. If ethnicity was reported, interactions between APOE4 carrier status, ethnicity, and outcomes were not investigated. Similarly, though sex was often reported, analysis of possible interactions between APOE4 carrier status, sex, and outcomes were also not performed. Subgroup analysis of sex and ethnicity in the meta-analysis conducted here was not possible as a result of the lack of ethnicity reporting (and a lack of diversity reported), and lack of disaggregated reporting of descriptive statistics. This is despite that the risk of carrying APOE4 does not affect all individuals equally [15, 19].

Furthermore, findings from TESTEX [37] demonstrated that study quality is lacking on some areas of design and reporting. When considering design, marks were often lost for not increasing the relative intensity of the exercise intervention. This is important as individuals adapt to the intensity of the exercise as early as 3–4 weeks into the exercise programme making the intervention less effective [37]. Additionally, marks were also frequently lost for monitoring of activity levels of control groups. When considering study reporting, marks were consistently lost on reporting of adherence and adverse events. In RCTs predominantly conducted in older adults at risk of dementia, the reporting of adverse events is important to be able to fully assess the risks and benefits of the intervention. Finally, the reporting of the exercise intervention was not sufficient in most studies as many failed to report exercise intensity defined by a quantitative measure. This makes it difficult to compare findings across RCTs to determine which exercise parameters are the most effective.

This systematic review and meta-analysis is primarily limited by a lack of data availability which resulted in the objective of the study not being fully addressed. A small number of published studies in this area meant that few were available for inclusion in the meta-analysis. Despite all included articles analysing data by APOE carrier status, few provided data segregated by APOE carrier status in the article, and requests for data by email were not often answered. This meant that few studies contributed to each outcome in the meta-analysis, and several planned analyses could not be conducted. Furthermore, the length of time to latest follow-up, where scores were taken from for use in the meta-analysis, varied between studies. This may have contributed to heterogeneity in the meta-analysis.

Moving forward, in addition to the improvements in quality of reporting previously discussed, future research may wish to ensure that studies are sufficiently powered to detect clinically meaningful differences between APOE4 carriers and non-carriers. This is important as analyses of APOE carrier status are typically secondary analyses and may be underpowered to detect clinically important differences.

## Conclusions

In conclusion, this systematic review and meta-analysis aimed to understand how APOE4 carrier status influences the physiological and cognitive response to exercise in RCTs. Findings showed that only very low- to moderate-quality evidence was available to address the research objective, and conclusive answers about the impact of APOE4 genotype on exercise intervention could not be made. Very low certainty evidence from the meta-analysis found limited evidence of difference between APOE4 carriers and non-carriers on measures of physical function. Future high-quality research is required to better understand if responses to exercise intervention differ between APOE4 carriers and non-carriers.

## Supplementary Information

Supplementary Material 1.

Supplementary Material 2.

Supplementary Material 3.

Supplementary Material 4.

## Data Availability

The dataset supporting the conclusions made in this article will be made available and provided in full on suitable request.

## Abbreviations

AD: Alzheimer’s disease
MCI: Mild cognitive impairment
APOE: Apolipoprotein E
PET: Positron emission topography
RCTs: Randomised controlled trials
PRISMA: Preferred Reporting Items for Systematic Reviews and Meta-Analyses
CASP: Critical Appraisal Skills Programme
TESTEX: The Tool for the Assessment of Study qualiTy and reporting in Exercise
ROB: Risk of Bias
SD: Standard deviation
SE: Standard error
ROB-ME: Risk of Bias due to Missing Evidence
CDR: Clinical Dementia Rating
MMSE: Mini Mental State Examination
V̇O_2_: Volume of Oxygen
BDNF: Blood-derived neurotrophic factor
MI: Moderate intensity
HI: High intensity
RBMT: Rivermead Behavioural Memory Test
WMS: Wechsler Memory Scale
SPPB: Short physical performance battery
ADL: Activities of daily living
HDL: High density lipoprotein
BMI: Body mass index
HRR: Heart rate reserve
WAIS: Wechsler Adult Intelligence Scale
ADAS-Cog: Alzheimer Disease Assessment Scale
LI: Low intensity
HR_max_: Heart rate max
CAIDE: Cardiovascular Risk Factors, Aging, and Incidence of Dementia
LTL: Relative leukocyte telomere length
NTB: Neuropsychological test battery
SF-36: Short Form Health Survey
MCS: Mental component score
PCS: Physical component score
CSF: Cerebrospinal fluid
