# Supplementary material for: The effect of the APOE4 genotype on physiological and cognitive health in randomised controlled trials with an exercise intervention: a systematic review and meta-analysis
Source: Trials. 2025 Jan 20;26:20. doi: 10.1186/s13063-024-08696-4 (PMC11744846; doi:10.1186/s13063-024-08696-4)
Supplement: Supplementary file 2 — Supplementary Material 2. [file 13063_2024_8696_MOESM2_ESM.docx]

| **Author** | **Exercise APOE4 Carrier** | **Exercise APOE4 non-Carrier** | **Control APOE4 Carrier** | **Control APOE4 non-Carrier** |
| --- | --- | --- | --- | --- |
| Allard et al., 2017 | Not reported | Not reported | Not reported | Not reported |
| Brown et al., 2021 | Not reported | Not reported | Not reported | Not reported |
| Cheng et al., 2014a | Not reported | Not reported | Not reported | Not reported |
| Cheng et al., 2014b | Not reported | Not reported | Not reported | Not reported |
| Eggermont et al., 2009a | Not reported | Not reported | Not reported | Not reported |
| Eggermont et al., 2009b | Not reported | Not reported | Not reported | Not reported |
| Galle et al., 2023 | Not reported | Not reported | Not reported | Not reported |
| Jensen et al., 2019 | - Symbol Digit Modalities Test (score in 90 minutes): 25.55 (SEM: 2.10) - Neuropsychiatric Inventory (score): 7.80 (SEM: 1.03) - Timed-Up-and-Go (s): 6.40 (SEM: 0.16) - Estimated VO_2_ Max (ml/kg/min): 28.60 (SEM: 0.98) - 400m walk (m): 293.04 (SEM: 7.18) - 10m walk (m/s): 1.34 (SEM: 0.02) - Dual task performance: Not reported | - Symbol Digit Modalities Test (score in 90 minutes): 24.49 (SEM: 2.84) - Neuropsychiatric Inventory (score): 10.10 (SEM: 1.54) - Timed-Up-and-Go (s): 6.76 (SEM: 0.38) - Estimated VO_2_ Max (ml/kg/min): 33.21 (SEM: 1.54) - 400m walk (m): 305.06 (SEM: 18.19) - 10m walk (m/s): 1.34 (SEM: 0.04) - Dual task performance: Not reported | - Symbol Digit Modalities Test (score in 90 minutes): 18.24 (SEM: 3.97) - Neuropsychiatric Inventory (score): 11.96 (SEM: 1.45) - Timed-Up-and-Go (s): 6.66 (SEM: 0.34) - Estimated VO_2_ Max (ml/kg/min): 26.89 (SEM: 1.22) - 400m walk (m): 307.63 (SEM: 9.50) - 10m walk (m/s): 1.37 (SEM: 0.03) - Dual task performance: Not reported | - Symbol Digit Modalities Test (score in 90 minutes): 26.05 (SEM: 3.35) - Neuropsychiatric Inventory (score): 11.49 (SEM: 2.40) - Timed-Up-and-Go (s): 6.44 (SEM: 0.31) - Estimated VO_2_ Max (ml/kg/min): 25.06 (SEM: 1.31) - 400m walk (m): 298.48 (SEM: 15.34) - 10m walk (m/s): 1.35 (SEM: 0.04) - Dual task performance: Not reported |
| Karssemeijer et al., 2019 | Not reported | Not reported | Not reported | Not reported |
| Lautenschlager et al., 2008 | Not reported | Not reported | Not reported | Not reported |
| Legault et al., 2011 | Not reported | Not reported | Not reported | Not reported |
| Sanders et al., 2020 | - 6 minute walk test (m): 296 (102) - Short Physical Performance Battery (score): 9.06 (1.99) - 6 Meter Walk Speed (m/s): 1.02 (0.29) - FICSIT-4 (score): 3.36 (1.41) - Timed-Up-And-Go (s): 13.3 (6.33) - Leg Strength (N): 206 (82.9) - MMSE (score): 18.6 (4.54) - Trail Making Test – A (s): 139 (60.1) - STROOP word (# correct): 46.4 (17.7) - STROOP colour (# correct): 37.3 (14.3) - STROOP colour-word (# correct): 13.7 (8.64) - STROOP interference quotient: 4.48 (3.78) - Digit Span Forwards (# correct): 6.56 (1.77) - Digit Span Backwards (# correct): 3.79 (1.34) - Visual Memory Span Forwards (# correct): 5.21 (1.67) - Verbal Memory Span Backwards (# correct): 4.34 (1.71) - Fluency (# correct): 21.6 (8.31) | - 6 minute walk test (m): 282 (91.0) - Short Physical Performance Battery (score): 8.87 (2.64) - 6 Meter Walk Speed (m/s): 0.95 (0.23) - FICSIT-4 (score): 3.22 (1.29) - Timed-Up-And-Go (s): 14.7 (6.94) - Leg Strength (N): 221 (107) - MMSE (score): 22.0 (4.50) - Trail Making Test – A (s): 116 (69.1) - STROOP word (# correct): 59.6 (20.8) - STROOP colour (# correct): 48.4 (16.6) - STROOP colour-word (# correct): 20.2 (11.5) - STROOP interference quotient: 2.78 (1.10) - Digit Span Forwards (# correct): 6.76 (1.88) - Digit Span Backwards (# correct): 4.19 (1.30) - Visual Memory Span Forwards (# correct): 5.25 (1.41) - Verbal Memory Span Backwards (# correct): 4.75 (1.74) - Fluency (# correct): 21.6 (8.86) | - 6 minute walk test (m): 249 (89.6) - Short Physical Performance Battery (score): 7.67 (1.56) - 6 Meter Walk Speed (m/s): 0.84 (0.21) - FICSIT-4 (score): 3.08 (1.00) - Timed-Up-And-Go (s): 15.9 (5.57) - Leg Strength (N): 208 (86.1) - MMSE (score): 20.3 (4.94) - Trail Making Test – A (s): 142 (54.8) - STROOP word (# correct): 51.2 (21.4) - STROOP colour (# correct): 41.4 (15.1) - STROOP colour-word (# correct): 18.0 (8.24) - STROOP interference quotient: 2.79 (1.49) - Digit Span Forwards (# correct): 6.83 (1.75) - Digit Span Backwards (# correct): 3.92 (1.73) - Visual Memory Span Forwards (# correct): 4.33 (1.97) - Verbal Memory Span Backwards (# correct): 4.08 (2.15) - Fluency (# correct): 19.1 (11.6) | - 6 minute walk test (m): 231 (87.6) - Short Physical Performance Battery (score): 7.57 (2.86) - 6 Meter Walk Speed (m/s): 0.76 (0.30) - FICSIT-4 (score): 2.99 (1.61) - Timed-Up-And-Go (s): 19.3 (7.92) - Leg Strength (N): 185 (51.3) - MMSE (score): 17.7 (6.33) - Trail Making Test – A (s): 160 (58.0) - STROOP word (# correct): 44.9 (19.3) - STROOP colour (# correct): 35.7 (15.6) - STROOP colour-word (# correct): 15.9 (9.33) - STROOP interference quotient: 3.16 (2.60) - Digit Span Forwards (# correct): 6.51 (2.04) - Digit Span Backwards (# correct): 4.12 (1.54) - Visual Memory Span Forwards (# correct): 4.61 (2.14) - Verbal Memory Span Backwards (# correct): 3.82 (1.90) - Fluency (# correct): 14.4 (7.67) |
| Sindi et al., 2021 | Not reported | Not reported | Not reported | Not reported |
| Solomon et al., 2018 | - NTB (total score; z score): 0.15 (0.72) - NTB (executive function; z score): 0.09 (0.75) - NTB (memory; z score): 0.25 (0.93) - NTB (processing speed; z score): 0.06 (0.82) - NTB (abbreviated memory; z score): 0.21 (0.92) | - NTB (total score; z score): 0.23 (0.65) - NTB (executive function; z score): 0.11 (0.70) - NTB (memory; z score): 0.40 (0.77) - NTB (processing speed; z score): 0.10 (0.86) - NTB (abbreviated memory; z score): 0.28 (0.80) | - NTB (total score; z score): 0.16 (0.70) - NTB (executive function; z score): 0.04 (0.74) - NTB (memory; z score): 0.29 (0.85) - NTB (processing speed; z score): 0.07 (0.93) - NTB (abbreviated memory; z score): 0.17 (0.87) | - NTB (total score; z score): 0.24 (0.68) - NTB (executive function; z score): 0.14 (0.72) - NTB (memory; z score): 0.40 (0.78) - NTB (processing speed; z score): 0.11 (0.89) - NTB (abbreviated memory; z score): 0.25 (0.79) |
| Solomon et al., 2021 | Not reported | Not reported | Not reported | Not reported |
| Stern et al., 2019 | - VO_2_max (ml/kg/min): 28.19 (9.87) - Processing speed composite (z score): 0.35 (0.87) - Episodic memory composite score (z score): 0.77 (0.79) - Working memory composite score (z score): 0.43 (0.49) - Language composite score (z score): -0.04 (0.55) - Attention composite score (z score): -0.05 (1.07) - Executive function composite score (z score): 0.14 (1.07) - BMI: 27.83 (6.45) - Activities of Daily Living (number of errors): 0.25 (0.45) - Cortical thickness: Not reported | - VO_2_max (ml/kg/min): 28.70 (6.36) - Processing speed composite (z score): 0.01 (0.79) - Episodic memory composite score (z score): 0.28 (0.91) - Working memory composite score (z score): 0.41 (0.69) - Language composite score (z score): 0.22 (0.96) - Attention composite score (z score): -0.65 (2.22) - Executive function composite score (z score): 0.38 (0.57) - BMI: 26.38 (4.76) - Activities of Daily Living (number of errors): 0.50 (0.73) - Cortical thickness: Not reported | - VO_2_max (ml/kg/min): 28.98 (10.06) - Processing speed composite (z score): 0.30 (0.62) - Episodic memory composite score (z score): 0.84 (0.86) - Working memory composite score (z score): 0.53 (0.73) - Language composite score (z score): 0.14 (0.85) - Attention composite score (z score): -0.59 (1.72) - Executive function composite score (z score): 0.45 (0.66) - BMI: 26.39 (5.07) - Activities of Daily Living (number of errors): 0.47 (0.80) - Cortical thickness: Not reported | - VO_2_max (ml/kg/min): 29.55 (6.67) - Processing speed composite (z score): 0.26 (0.89) - Episodic memory composite score (z score): 0.45 (0.82) - Working memory composite score (z score): 0.40 (0.59) - Language composite score (z score): 0.42 (1.04) - Attention composite score (z score): -0.43 (1.70) - Executive function composite score (z score): 0.32 (0.79) - BMI: 26.76 (4.64) - Activities of Daily Living (number of errors): 0.37 (0.72) - Cortical thickness: Not reported |
| Stonnington et al., 2020 | Not reported | Not reported | Not reported | Not reported |
| Vidoni et al., 2021 | Not reported | Not reported | Not reported | Not reported |
| Yu et al., 2022 | Not reported | Not reported | Not reported | Not reported |
