# Supplementary figures and images for: The effect of the APOE4 genotype on physiological and cognitive health in randomised controlled trials with an exercise intervention: a systematic review and meta-analysis
Source: Trials. 2025 Jan 20;26:20. doi: 10.1186/s13063-024-08696-4 (PMC11744846; doi:10.1186/s13063-024-08696-4)

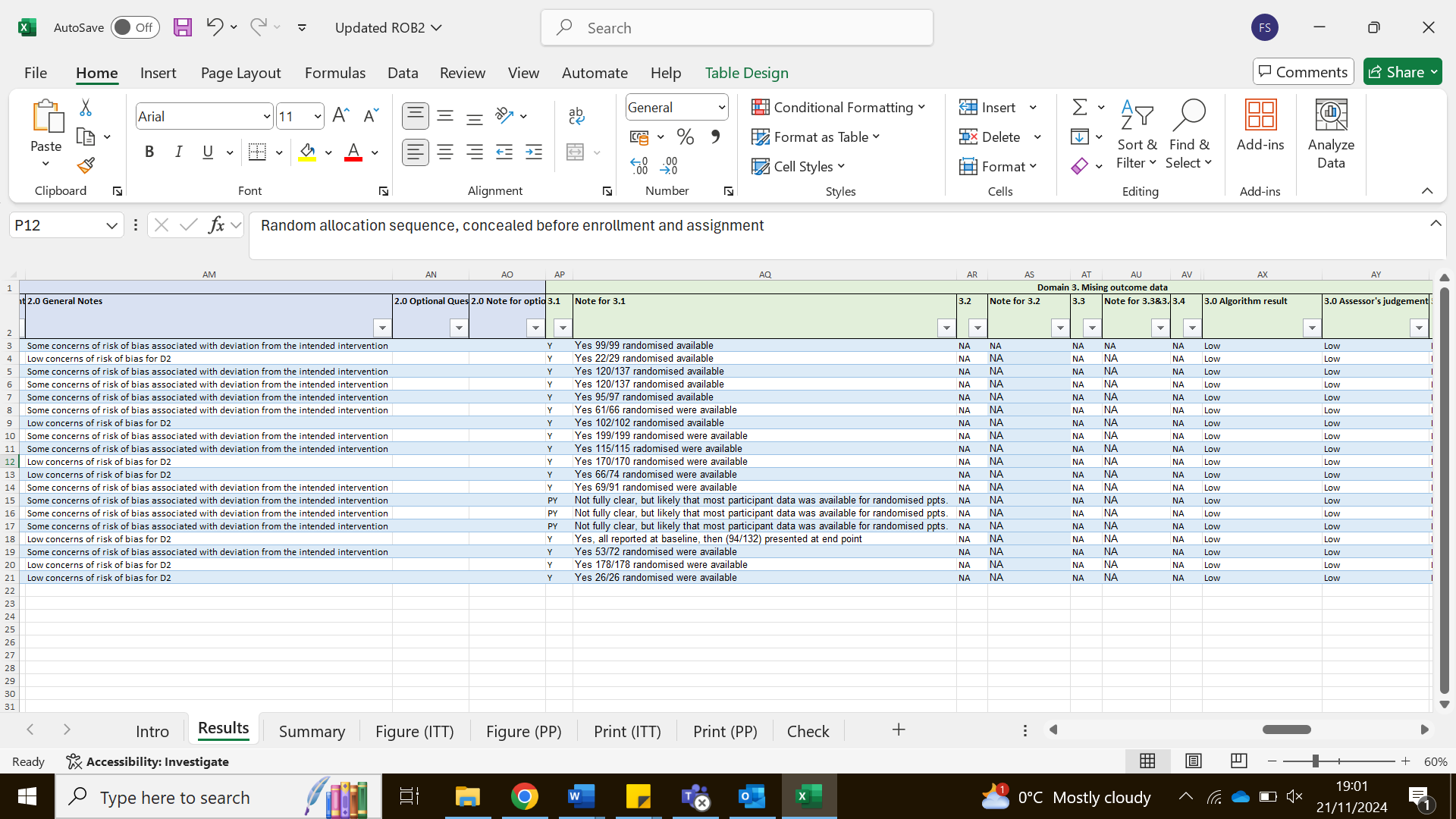

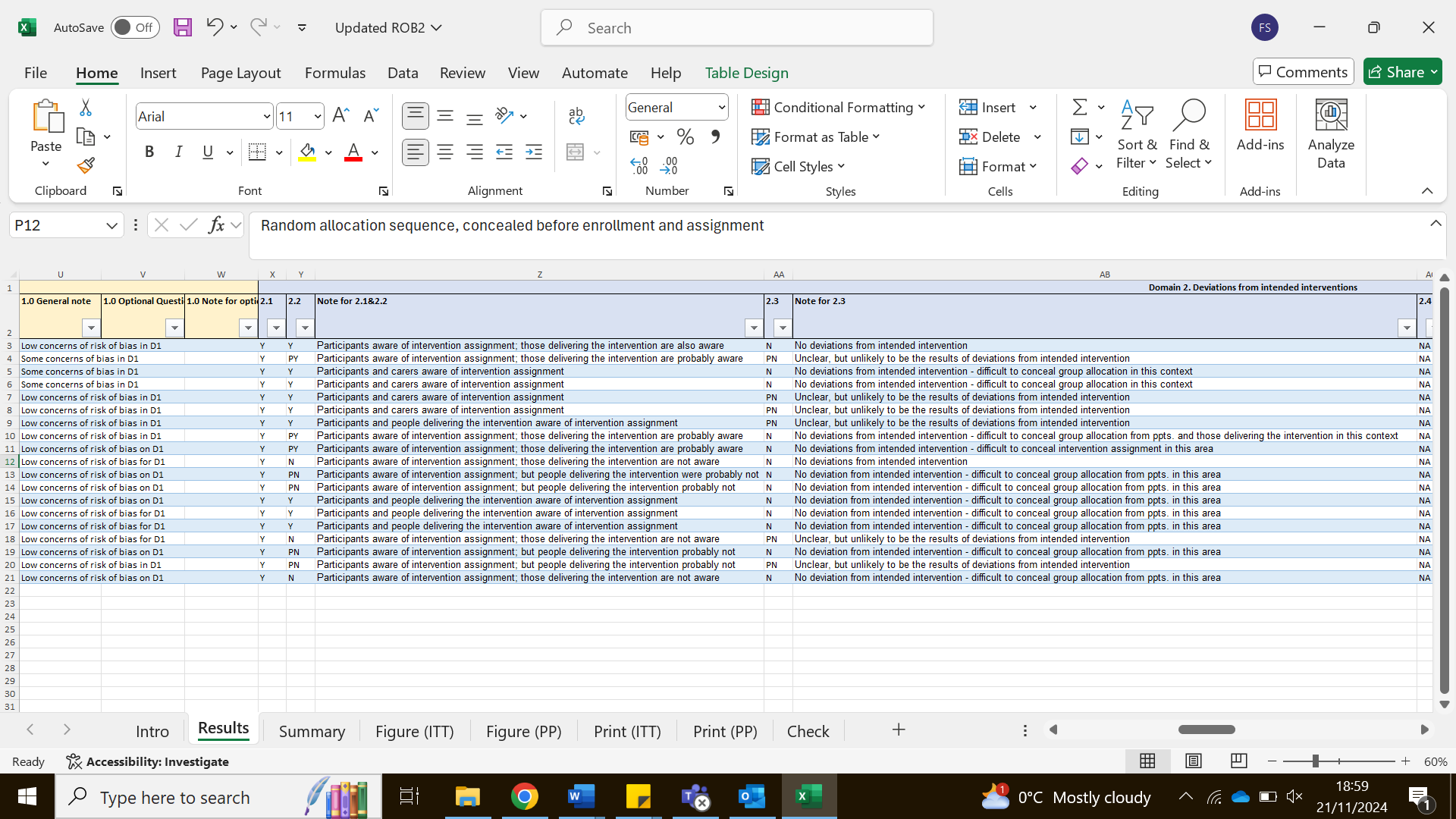

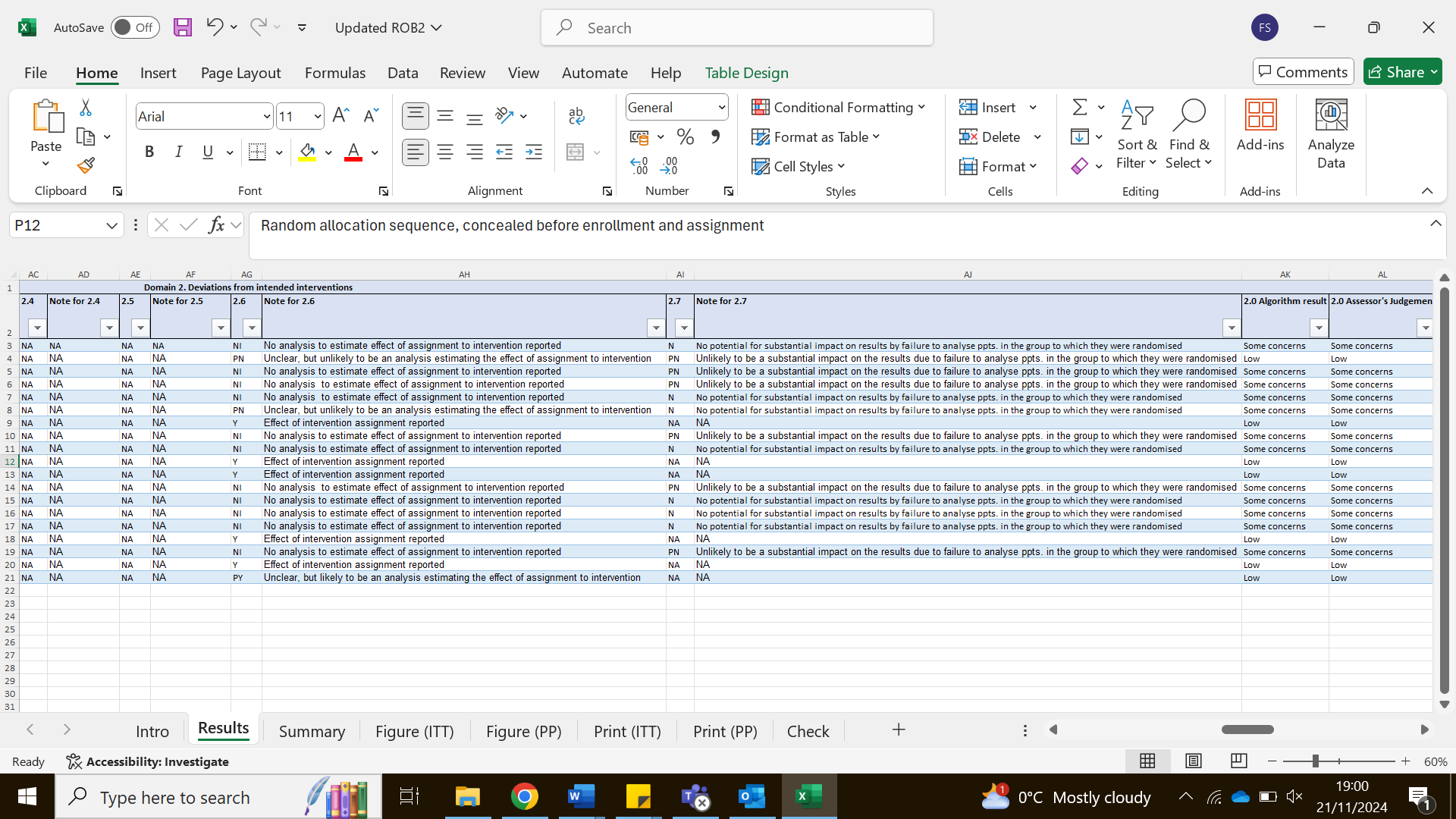

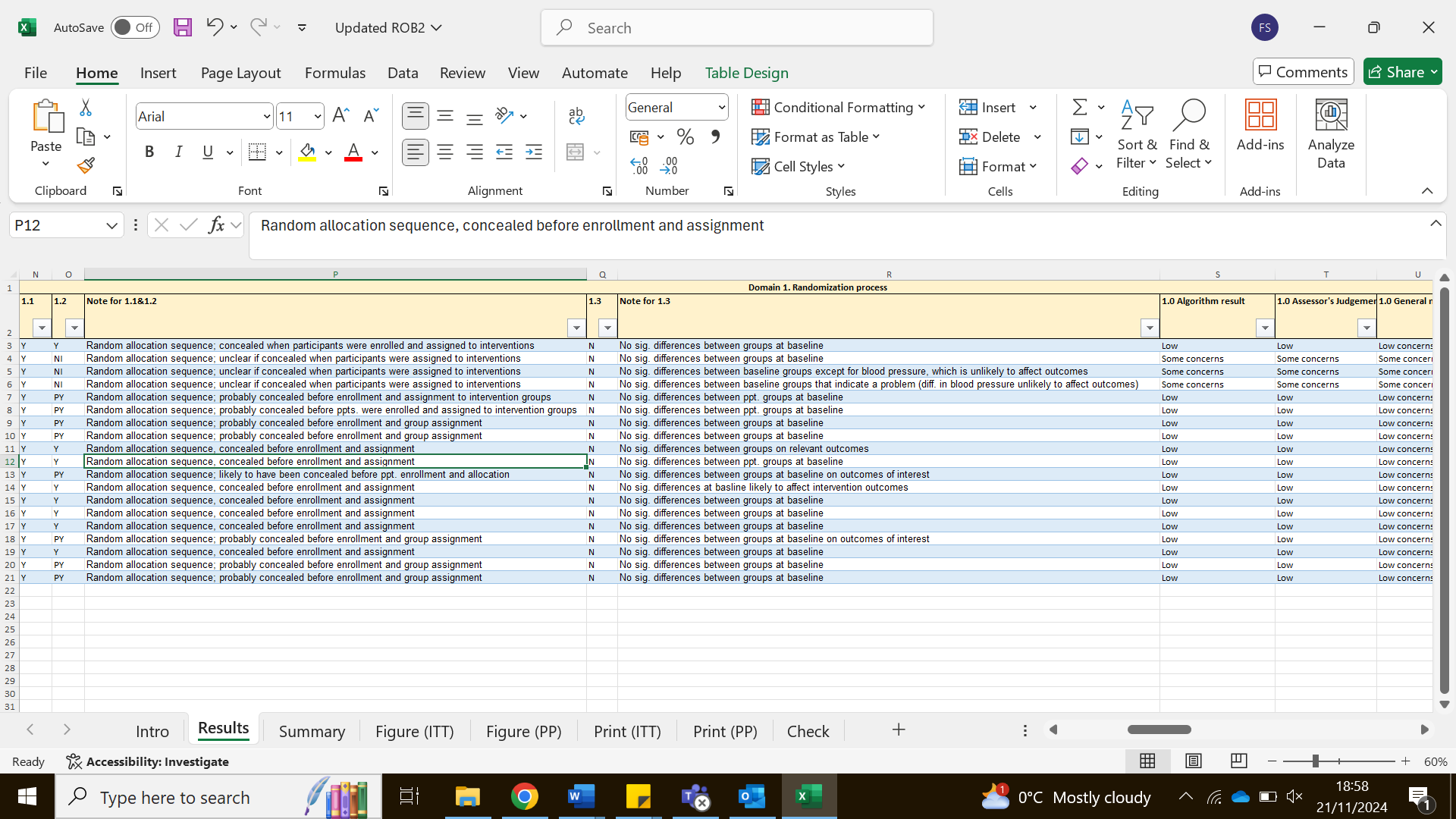

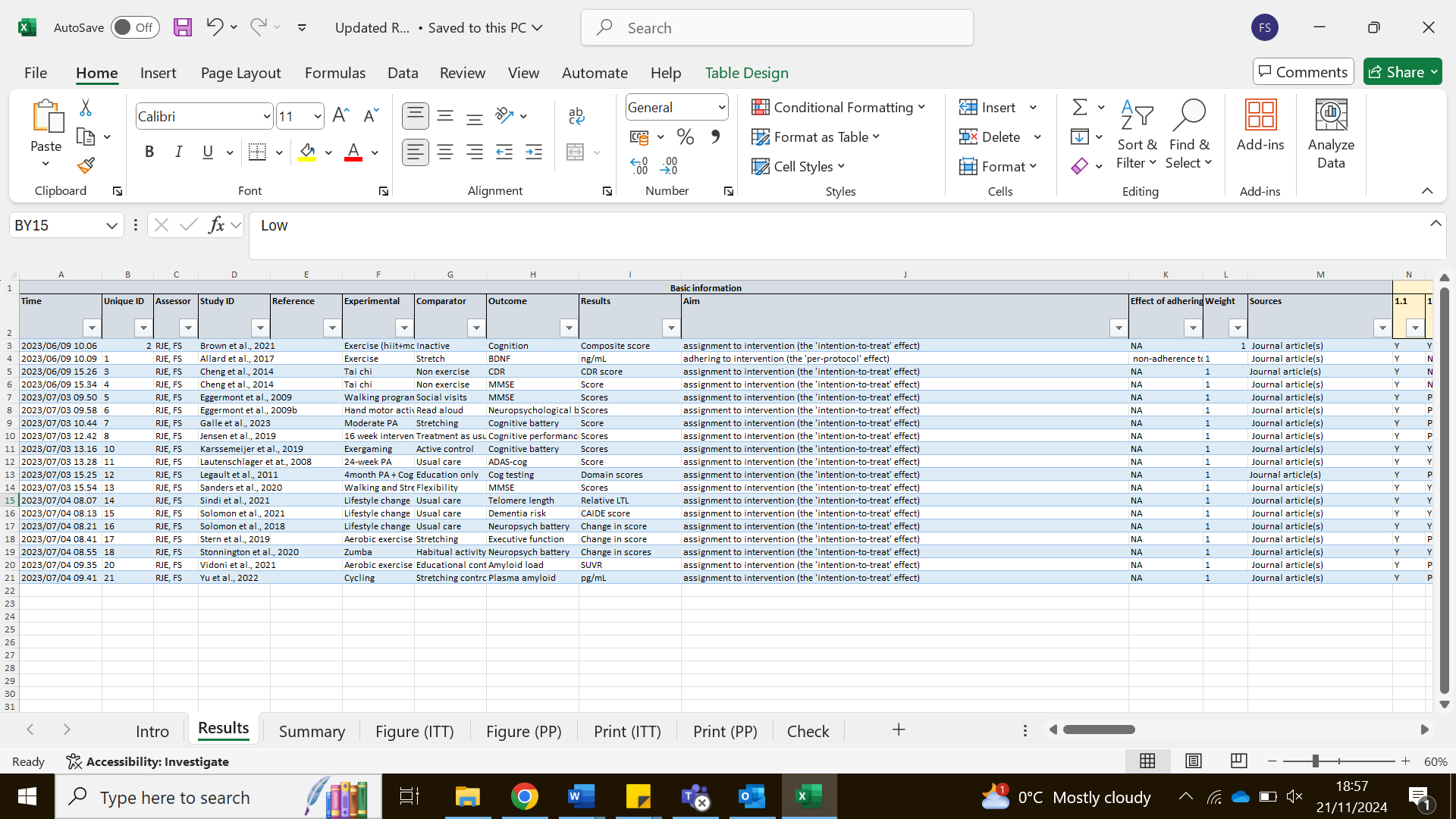


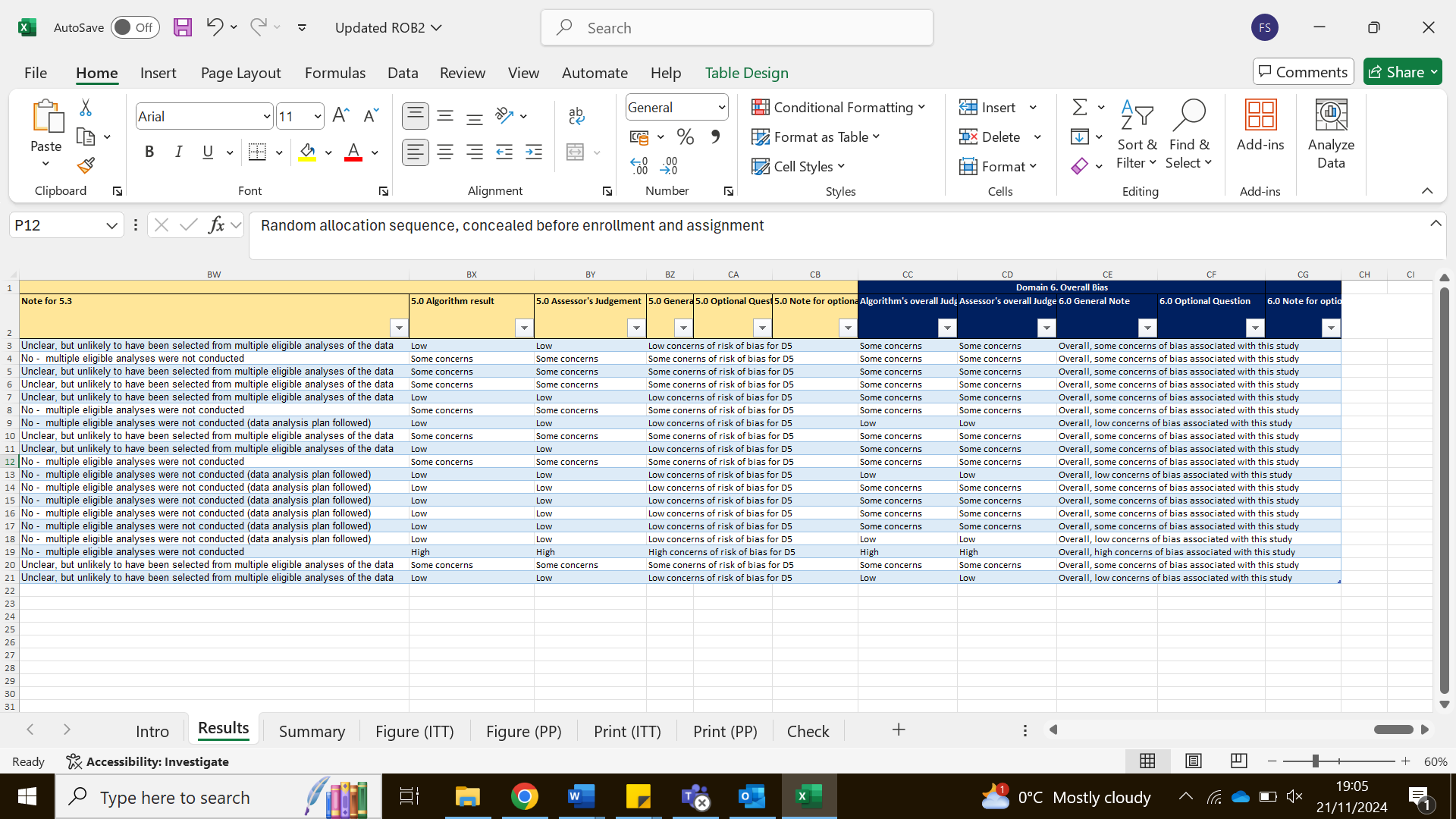

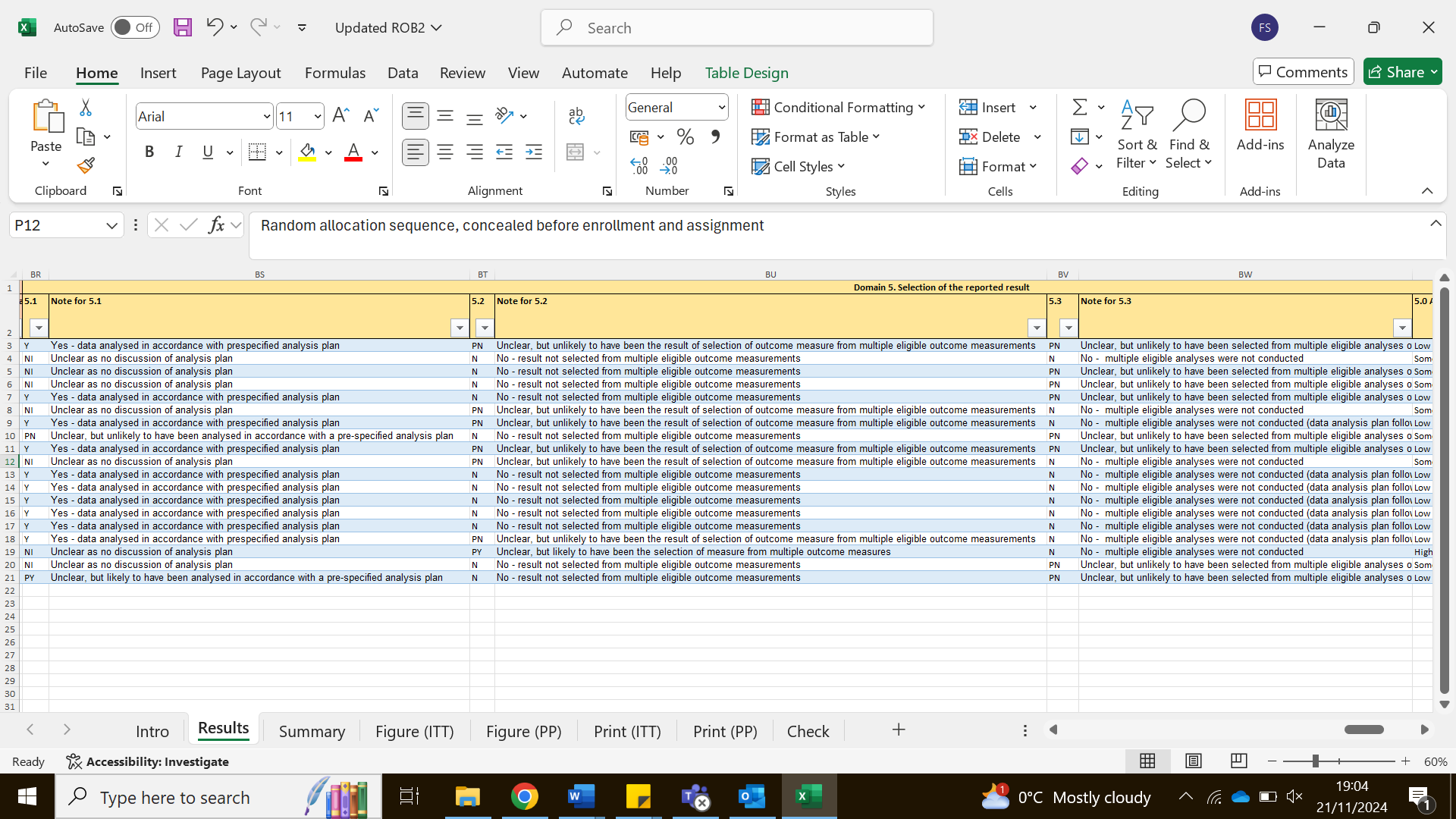

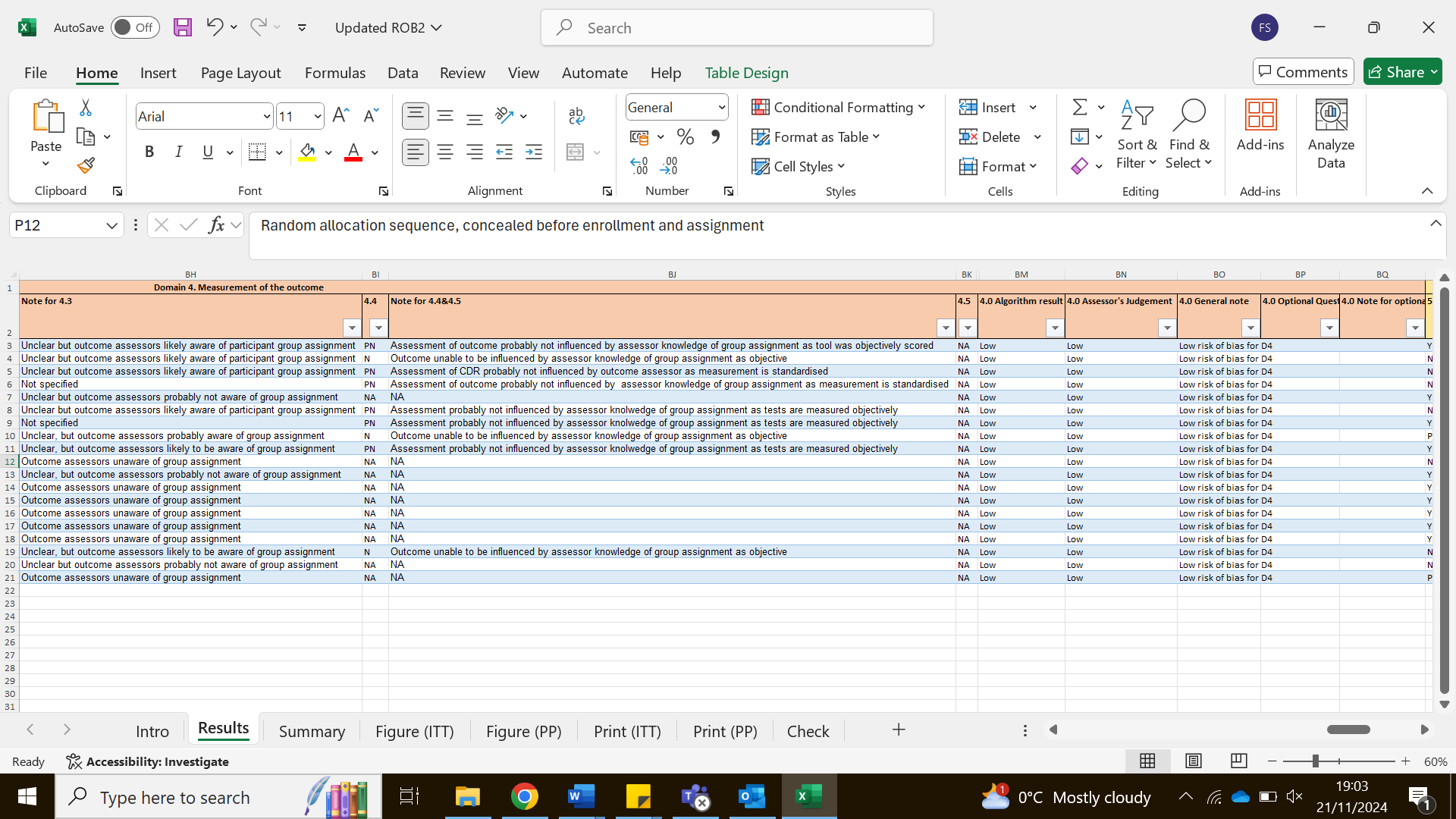

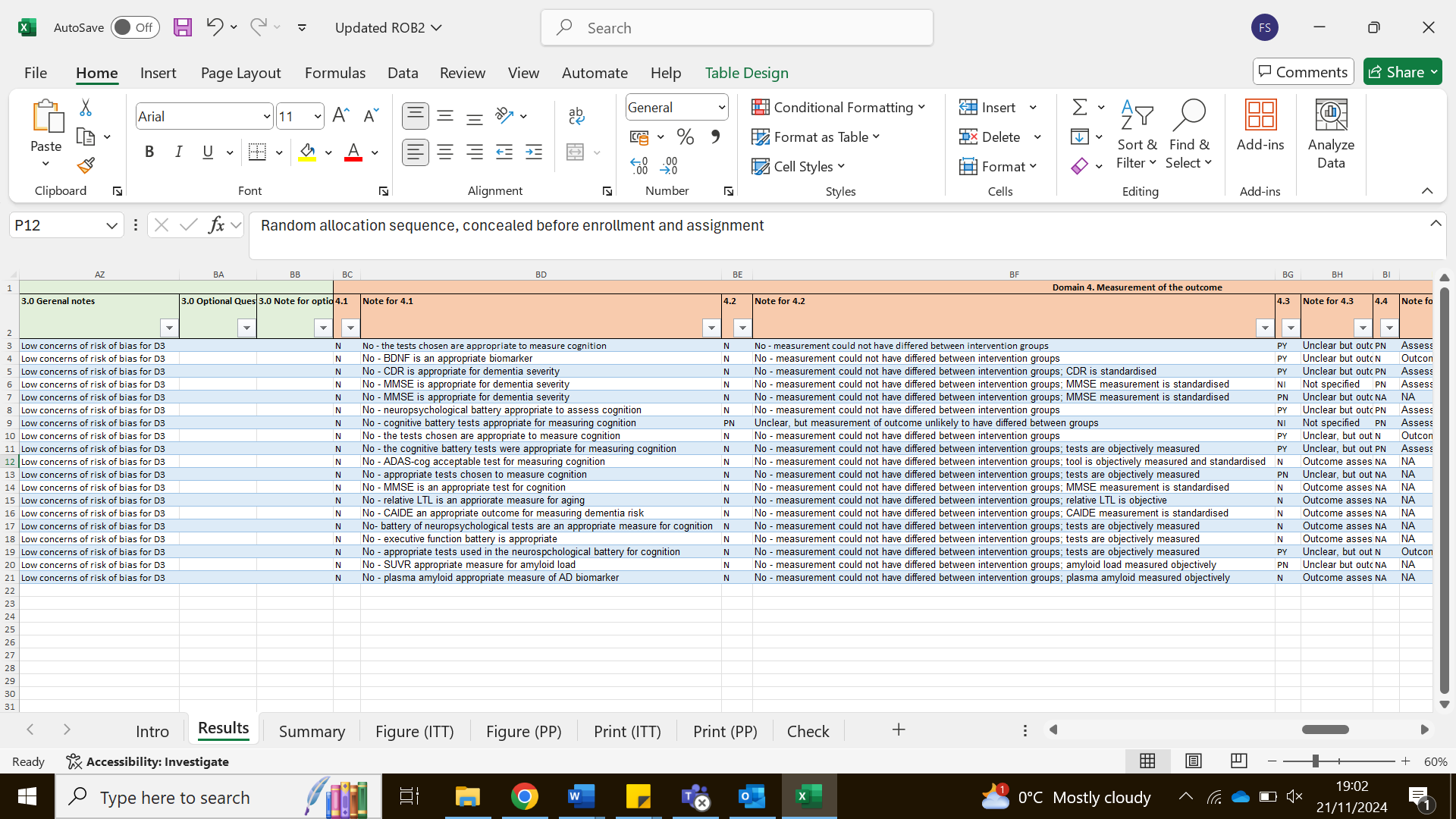

Supplement: Supplementary file 3 — Supplementary Material 3. [file 13063_2024_8696_MOESM3_ESM.docx]
