# Supplementary material for: The effect of the APOE4 genotype on physiological and cognitive health in randomised controlled trials with an exercise intervention: a systematic review and meta-analysis
Source: Trials. 2025 Jan 20;26:20. doi: 10.1186/s13063-024-08696-4 (PMC11744846; doi:10.1186/s13063-024-08696-4)
Supplement: Supplementary file 4 — Supplementary Material 4. [file 13063_2024_8696_MOESM4_ESM.docx]

**Tool for assessing Risk Of Bias due to Missing Evidence in a meta-analysis (ROB-ME)**

**TEMPLATE FOR COMPLETION**

**Version 1 October 2023**

ROB-ME Development Group:

 Matthew J Page, Jonathan AC Sterne, Isabelle Boutron, Asbjørn Hróbjartsson, Jamie J Kirkham, Tianjing Li, Andreas Lundh, Evan Mayo-Wilson, Joanne E McKenzie, Lesley A Stewart, Alex J Sutton, Lisa Bero, Adam G Dunn, Kerry Dwan, Roy G Elbers, Raju Kanukula, Joerg J Meerpohl, Erick H Turner, Julian PT Higgins

**Correspondence to:** Dr. Matthew Page, Methods in Evidence Synthesis Unit, School of Public Health and Preventive Medicine, Monash University, 553 St Kilda Road, Melbourne, Victoria, 3004, Australia. Telephone: +61 9903 0248. Email address: [matthew.page@monash.edu](about:blank)


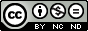


This work is licensed under a [Creative Commons Attribution-NonCommercial-NoDerivatives 4.0 International License](about:blank).

| **Step 1. Select and define meta-analyses that will be assessed for risk of bias due to missing evidence** | | |
| --- | --- | --- |
| **Meta-analysis ID** | **Specify the PICO for all meta-analyses that will be assessed for risk of bias. For example:**  ***Participants:*** *People with shoulder pain*  ***Intervention:*** *Ibuprofen*  ***Comparator:*** *Placebo*  ***Outcome:*** *Pain intensity at short-term (0-12 weeks)*  **Add/delete rows where necessary** | **For each meta-analysis, specify which study designs and results were eligible for inclusion, indicating whether the meta-analysis was restricted to particular:**   - **study designs, and;** - **outcome definitions (e.g. measures, metrics, time points), and;** - **methods of analysis (e.g. analysis populations, crude or adjusted estimates).**   **If such information is reported elsewhere in the systematic review, either indicate the relevant section of the review or copy the information here.**  **For example:**  ***Eligible study designs:*** *Randomized trials*  ***Eligible outcome definitions:*** *Pain scores measured using any scale; up to 12 weeks post-randomization*  ***Eligible methods of analysis:*** *Analyses of change from baseline values; intention-to-treat analysis sample; analyses adjusted for covariates* |
| 1 | **Participants:** People with known APOE genotype (regardless of cognitive ability)  **Intervention:** Exercise  **Comparator:** Non-exercise  **Outcome:** VO_2_ Max | **Eligible study designs:** Randomised trials  **Eligible outcome definitions:** VO_2_ max measured by cycling on an exercise bike; collected from latest timepoint of data collection  **Eligible methods of analysis:** Analyses of change from baseline values; intention-to-treat analysis sample |
| 2 | **Participants:** People with known APOE genotype (regardless of cognitive ability)  **Intervention:** Exercise  **Comparator:** Non-exercise  **Outcome:** Walking Endurance | **Eligible study designs:** Randomised trials  **Eligible outcome definitions:** Walking endurance measured by 400m or 6-minute walk test; collected from latest timepoint of data collection  **Eligible methods of analysis:** Analyses of change from baseline values; intention-to-treat analysis sample |
| 3 | **Participants:** People with known APOE genotype (regardless of cognitive ability)  **Intervention:** Exercise  **Comparator:** Non-exercise  **Outcome:** Gait speed | **Eligible study designs:** Randomised trials  **Eligible outcome definitions:** Gait speed measured by 6m or 10m walk test; collected from latest timepoint of data collection  **Eligible methods of analysis:** Analyses of change from baseline values; intention-to-treat analysis sample |
| 4 | **Participants:** People with known APOE genotype (regardless of cognitive ability)  **Intervention:** Exercise  **Comparator:** Non-exercise  **Outcome:** Mobility | **Eligible study designs:** Randomised trials  **Eligible outcome definitions:** Mobility measured by any scale; collected from latest timepoint of data collection  **Eligible methods of analysis:** Analyses of change from baseline values; intention-to-treat analysis sample |

| **Step 2. Determine which studies meeting the inclusion criteria for the meta-analyses have missing results** |
| --- |
| **For each study meeting the inclusion criteria for one or more of the meta-analyses, assemble available sources of information about the study. This might include the trials register entry (e.g. at ClinicalTrials.gov), study protocol, statistical analysis plan, reports of results of the study (e.g. journal article, clinical study report), or information obtained directly from the study authors or sponsor (e.g. data files supplied).**  **Then compare results available with all available information about what outcomes were measured. If study plans are available (e.g. trials register entry, protocol), compare results available with details of the pre-specified outcomes, to identify any outcomes with no results reported. It might be helpful to construct a matrix for each eligible study that lists all outcomes described in the study plans and records whether results were available for each. If no study plans are available, cross-check the methods and results sections against one another to identify any outcomes with no results reported, or results reported incompletely.**  **Then complete the Results Matrix below to indicate (using the symbols in the Key below) whether study results are available for inclusion in each meta-analysis to be assessed for risk of bias.**  **Also specify the total number of participants analysed for an indication of the likely weight of each study in the meta-analysis.**  **Key for Results Matrix**   \| ✔ \| A study result is available for inclusion in the meta-analysis. \| \| --- \| --- \| \| ~ \| No study result is available for inclusion in the meta-analysis, for a reason unrelated to the P value, magnitude or direction of the result. \| \| ? \| Unclear whether an eligible study result was generated. \| \| X \| No study result is available for inclusion in the meta-analysis, likely because of the P value, magnitude or direction of the result generated.  *Optional: Record any information known about the results (if available), such as the direction of effect (e.g. Favours intervention / Favours control), the statistical significance of the result (e.g. P > 0.05), or narrative descriptions (e.g. “No difference”).* \|     **Example of a completed Results Matrix**   \| **Study ID** \| **Source(s) used** \| **Number of participants analysed** \| **Result available for inclusion in Meta-analysis 1** \| **Result available for inclusion in Meta-analysis 2** \| **Result available for inclusion in Meta-analysis 3** \| **Result available for inclusion in Meta-analysis 4** \| \| --- \| --- \| --- \| --- \| --- \| --- \| --- \| \| Smith 2000 \| PMID: XXXXXXXX \| 455 \| ✔ \| X \| ✔ \| ? \| \| Nyqvist 2017 \| None (not published) \| 67 \| X \| X \| ~ \| ~ \| \| Stylianos 2019 \| PMID: XXXXXXXX \| 87 \| ? \| ✔ \| ✔ \| X \| \| Hozo 2014 \| PMID: XXXXXXXX \| 145 \| X \| ✔ \| X \| ✔ \| \| MacIntyre 2020 \| NCTXXXXXXXX \| 280 \| ~ \| ✔ \| X \| ~ \|   **Results Matrix (add/delete rows and columns where necessary)**   \| **Study ID*** \| **Source(s) used** \| **Number of participants analysed**** \| **Result available for inclusion in Meta-analysis 1** \| **Result available for inclusion in Meta-analysis 2** \| **Result available for inclusion in Meta-analysis 3** \| **Result available for inclusion in Meta-analysis 4** \| \| --- \| --- \| --- \| --- \| --- \| --- \| --- \| \| Jensen 2019 \| Journal article; clinical trials.gov \| 200 \| ✔ \| ✔ \| ✔ \| ✔ \| \| Sanders 2020 \| Journal article \| 69 \| ~ \| ✔ \| ✔ \| ✔ \| \| Stern 2019 \| Journal article; clinical trials.gov \| 132 \| ✔ \| ~ \| ~ \| ~ \|  \| *List all studies meeting the inclusion criteria for the meta-analyses, regardless of whether a report of the results is available. Ideally, also include in the matrix any study identified that did not measure the outcome (but met all other inclusion criteria) and any study that was excluded from the systematic review *only* because it had no usable outcome data (but met all other inclusion criteria).  **If it is not clear how many participants were analysed, record the total number of participants assigned to the relevant intervention and control groups  **Provide any relevant information to support responses**   \| Clinical trial registries showed pre-registration of outcomes to be investigated; demonstrating that outcomes were not chosen selectively; and were unavailable due to archiving, there was no evidence of non-reporting of result due to p value, magnitude, or direction of result (more likely due to this outcome not being measured) \| \| --- \| \| \| --- \| --- \| |

| **Step 3. Consider the potential for missing studies across the systematic review** |
| --- |
| **Answer the following questions to determine whether circumstances indicate potential for some eligible studies not being identified because of the P value, magnitude or direction of the results generated. Answer these questions once, in relation to the systematic review as a whole.**   \| **Question** \| **Response options** \| \| --- \| --- \| \| **3.1. Were prospectively registered studies or studies identified for a prospective meta-analysis the only type of study eligible for inclusion in the review?** \| Y / N \| \| **3.2. If N to 3.1: Would you expect every eligible study to be identifiable regardless of its results?** \| NA / Y / PY / PN / N \| \| **3.3. If Y/PY to 3.2: Were you likely to have found all eligible studies regardless of their results?** \| NA / Y / PY / PN / N \|   Y: ‘Yes’; PY: ‘Probably yes’; PN: ‘Probably no’; N: ‘No’; NA: ‘Not applicable’.  **Check the box below if the response to 3.1 was ‘No’ and the response to 3.2 or 3.3 was ‘No / Probably no’**  X Circumstances indicate potential for some eligible studies not being identified because of the P value, magnitude or direction of the results generated  **Provide any relevant information to support responses**   \| The systematic review was not limited to clinical trials, and there was no criteria of pre-registration for the review, so some studies were eligible for review that were not pre-registered. \| \| --- \| |

**Step 4. Assess risk of bias due to missing evidence in a meta-analysis (complete for each meta-analysis)**

Responses underlined in green are potential markers for low risk of bias, and responses in red are potential markers for a risk of bias.

| **Details of the meta-analysis being assessed for risk of bias** | | |
| --- | --- | --- |
| **Specify the meta-analysis** | Random-effects meta-analysis of the effect of exercise vs non-exercise on VO_2_ max | |
| **Specify the meta-analysis result (e.g. summary effect estimate and 95% CI)** | Std. mean difference = 0.23 [95% CI] [-0.12, 0.58] | |
| **Specify the number of included studies and participants** | 2 studies (268 participants) | |
| **Risk of bias assessment** | | |
| **Signalling questions** | **Comments** | **Response options** |
| ***The following questions relate to the within-study assessment of non-reporting bias (‘known unknowns’)*** | | |
| **4.1. Of the studies identified, was there any for which no result was available for inclusion in the meta-analysis, likely because of the P value, magnitude or direction of the result generated (refer to Step 2)?** |  | Y / N |
| **4.2. If Y to 4.1: Is it likely that there would be a notable change to the summary effect estimate if the omitted results had been included?** |  | NA / Y / PY / PN / N / NI |
| **4.3. Of the studies identified, was there any for which it was unclear whether an eligible result was generated (refer to Step 2)?** |  | Y / N |
| **4.4. If Y to 4.3: Is it likely that there would be a notable change to the summary effect estimate if the potentially omitted results had been included?** |  | NA / Y / PY / PN / N / NI |
| ***The following questions relate to the across-study assessment of non-reporting bias (‘unknown unknowns’)*** | | |
| **4.5 Do circumstances (identified in Step 3) indicate potential for some eligible studies not being identified because of the P value, magnitude or direction of the results generated?** |  | Y / N |
| **4.6. If Y to 4.5: Is it likely that studies not identified had results that were eligible for inclusion in the meta-analysis?** |  | NA / Y / PY / PN / N |
| **4.7. If Y to 4.1, 4.3 or 4.5: Does the pattern of observed study results suggest that the meta-analysis is likely to be missing results that were systematically different (in terms of P value, magnitude or direction) from those observed?** |  | NA / Y / PY / PN / N |
| **4.8. If Y/PY/NI to 4.2, 4.4, 4.6 or 4.7: Did sensitivity analyses suggest that the summary effect estimate was biased due to missing results?** |  | NA / Y / PY / PN / N |
| **Risk of bias judgement** |  | Low / High / Some concerns |
| Optional: What is the predicted direction of bias for this meta-analysis? |  | Favours experimental / Favours comparator / Towards null /Away from null / Unpredictable |

Y: ‘Yes’; PY: ‘Probably yes’; PN: ‘Probably no’; N: ‘No’; NI: ‘No information’; NA: ‘Not applicable’.

| **Details of the meta-analysis being assessed for risk of bias** | | |
| --- | --- | --- |
| **Specify the meta-analysis** | Random-effects meta-analysis of the effect of exercise vs non-exercise on walking endurance | |
| **Specify the meta-analysis result (e.g. summary effect estimate and 95% CI)** | Std. mean difference = 0.14 [95% CI] [-0.24, 0.52] | |
| **Specify the number of included studies and participants** | 2 studies (268 participants) | |
| **Risk of bias assessment** | | |
| **Signalling questions** | **Comments** | **Response options** |
| ***The following questions relate to the within-study assessment of non-reporting bias (‘known unknowns’)*** | | |
| **4.1. Of the studies identified, was there any for which no result was available for inclusion in the meta-analysis, likely because of the P value, magnitude or direction of the result generated (refer to Step 2)?** |  | Y / N |
| **4.2. If Y to 4.1: Is it likely that there would be a notable change to the summary effect estimate if the omitted results had been included?** |  | NA / Y / PY / PN / N / NI |
| **4.3. Of the studies identified, was there any for which it was unclear whether an eligible result was generated (refer to Step 2)?** |  | Y / N |
| **4.4. If Y to 4.3: Is it likely that there would be a notable change to the summary effect estimate if the potentially omitted results had been included?** |  | NA / Y / PY / PN / N / NI |
| ***The following questions relate to the across-study assessment of non-reporting bias (‘unknown unknowns’)*** | | |
| **4.5 Do circumstances (identified in Step 3) indicate potential for some eligible studies not being identified because of the P value, magnitude or direction of the results generated?** |  | Y / N |
| **4.6. If Y to 4.5: Is it likely that studies not identified had results that were eligible for inclusion in the meta-analysis?** |  | NA / Y / PY / PN / N |
| **4.7. If Y to 4.1, 4.3 or 4.5: Does the pattern of observed study results suggest that the meta-analysis is likely to be missing results that were systematically different (in terms of P value, magnitude or direction) from those observed?** |  | NA / Y / PY / PN / N |
| **4.8. If Y/PY/NI to 4.2, 4.4, 4.6 or 4.7: Did sensitivity analyses suggest that the summary effect estimate was biased due to missing results?** |  | NA / Y / PY / PN / N |
| **Risk of bias judgement** |  | Low / High / Some concerns |
| Optional: What is the predicted direction of bias for this meta-analysis? |  | Favours experimental / Favours comparator / Towards null /Away from null / Unpredictable |

| **Details of the meta-analysis being assessed for risk of bias** | | |
| --- | --- | --- |
| **Specify the meta-analysis** | Random-effects meta-analysis of the effect of exercise vs non-exercise on gait speed | |
| **Specify the meta-analysis result (e.g. summary effect estimate and 95% CI)** | Std. mean difference = 0.22 [95% CI] [-0.22, 0.66] | |
| **Specify the number of included studies and participants** | 2 studies (268 participants) | |
| **Risk of bias assessment** | | |
| **Signalling questions** | **Comments** | **Response options** |
| ***The following questions relate to the within-study assessment of non-reporting bias (‘known unknowns’)*** | | |
| **4.1. Of the studies identified, was there any for which no result was available for inclusion in the meta-analysis, likely because of the P value, magnitude or direction of the result generated (refer to Step 2)?** |  | Y / N |
| **4.2. If Y to 4.1: Is it likely that there would be a notable change to the summary effect estimate if the omitted results had been included?** |  | NA / Y / PY / PN / N / NI |
| **4.3. Of the studies identified, was there any for which it was unclear whether an eligible result was generated (refer to Step 2)?** |  | Y / N |
| **4.4. If Y to 4.3: Is it likely that there would be a notable change to the summary effect estimate if the potentially omitted results had been included?** |  | NA / Y / PY / PN / N / NI |
| ***The following questions relate to the across-study assessment of non-reporting bias (‘unknown unknowns’)*** | | |
| **4.5 Do circumstances (identified in Step 3) indicate potential for some eligible studies not being identified because of the P value, magnitude or direction of the results generated?** |  | Y / N |
| **4.6. If Y to 4.5: Is it likely that studies not identified had results that were eligible for inclusion in the meta-analysis?** |  | NA / Y / PY / PN / N |
| **4.7. If Y to 4.1, 4.3 or 4.5: Does the pattern of observed study results suggest that the meta-analysis is likely to be missing results that were systematically different (in terms of P value, magnitude or direction) from those observed?** |  | NA / Y / PY / PN / N |
| **4.8. If Y/PY/NI to 4.2, 4.4, 4.6 or 4.7: Did sensitivity analyses suggest that the summary effect estimate was biased due to missing results?** |  | NA / Y / PY / PN / N |
| **Risk of bias judgement** |  | Low / High / Some concerns |
| Optional: What is the predicted direction of bias for this meta-analysis? |  | Favours experimental / Favours comparator / Towards null /Away from null / Unpredictable |

| **Details of the meta-analysis being assessed for risk of bias** | | |
| --- | --- | --- |
| **Specify the meta-analysis** | Random-effects meta-analysis of the effect of exercise vs non-exercise on mobility | |
| **Specify the meta-analysis result (e.g. summary effect estimate and 95% CI)** | Std. mean difference = -0.35 [95% CI] [-1.41, 0.71] | |
| **Specify the number of included studies and participants** | 2 studies (268 participants) | |
| **Risk of bias assessment** | | |
| **Signalling questions** | **Comments** | **Response options** |
| ***The following questions relate to the within-study assessment of non-reporting bias (‘known unknowns’)*** | | |
| **4.1. Of the studies identified, was there any for which no result was available for inclusion in the meta-analysis, likely because of the P value, magnitude or direction of the result generated (refer to Step 2)?** |  | Y / N |
| **4.2. If Y to 4.1: Is it likely that there would be a notable change to the summary effect estimate if the omitted results had been included?** |  | NA / Y / PY / PN / N / NI |
| **4.3. Of the studies identified, was there any for which it was unclear whether an eligible result was generated (refer to Step 2)?** |  | Y / N |
| **4.4. If Y to 4.3: Is it likely that there would be a notable change to the summary effect estimate if the potentially omitted results had been included?** |  | NA / Y / PY / PN / N / NI |
| ***The following questions relate to the across-study assessment of non-reporting bias (‘unknown unknowns’)*** | | |
| **4.5 Do circumstances (identified in Step 3) indicate potential for some eligible studies not being identified because of the P value, magnitude or direction of the results generated?** |  | Y / N |
| **4.6. If Y to 4.5: Is it likely that studies not identified had results that were eligible for inclusion in the meta-analysis?** |  | NA / Y / PY / PN / N |
| **4.7. If Y to 4.1, 4.3 or 4.5: Does the pattern of observed study results suggest that the meta-analysis is likely to be missing results that were systematically different (in terms of P value, magnitude or direction) from those observed?** |  | NA / Y / PY / PN / N |
| **4.8. If Y/PY/NI to 4.2, 4.4, 4.6 or 4.7: Did sensitivity analyses suggest that the summary effect estimate was biased due to missing results?** |  | NA / Y / PY / PN / N |
| **Risk of bias judgement** |  | Low / High / Some concerns |
| Optional: What is the predicted direction of bias for this meta-analysis? |  | Favours experimental / Favours comparator / Towards null /Away from null / Unpredictable |


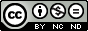


This work is licensed under a [Creative Commons Attribution-NonCommercial-NoDerivatives 4.0 International License](about:blank).
